# Healthcare Occupations, Suicides, and Suicide Attempts: A Cohort Study Based on the Working Population in Sweden

**DOI:** 10.1111/acps.70018

**Published:** 2025-07-20

**Authors:** Alicia Nevriana, Emma Brulin, Tomas Hemmingsson, Melody Almroth, Kuan‐Yu Pan, Theo Bodin, Katarina Kjellberg, Daniel Falkstedt

**Affiliations:** ^1^ Institute of Environmental Medicine, Karolinska Institutet Stockholm Sweden; ^2^ Department of Public Health Sciences Stockholm University Stockholm Sweden; ^3^ Centre for Occupational and Environmental Medicine, Region Stockholm Stockholm Sweden

**Keywords:** cohort, health personnel, suicide

## Abstract

**Introduction:**

Many studies have examined physicians' risk of suicide, but studies of other healthcare occupations have been fewer. Suicide attempts have also rarely been studied. We aimed to determine the risks of suicide and suicide attempts among healthcare workers in comparison with non‐healthcare workers, according to occupational qualification level.

**Methods:**

This population‐based cohort study linking Swedish national registers included 243,183 healthcare workers in high‐qualified occupations (e.g., physicians); 1,789,076 workers in other high‐qualified occupations; 514,726 healthcare workers in low‐qualified occupations (e.g., assistant nurses); and 2,026,890 workers in low‐qualified occupations residing in Sweden in 2005 and followed them until the latest December 31, 2020. We estimated adjusted hazard ratios (aHR) for suicide and first suicide attempt.

**Results:**

Compared to non‐healthcare workers, higher risks for suicide were observed for several healthcare occupations, primarily those working with patient care (e.g., aHR physicians 1.57, 95% CI: 1.23–2.00, registered nurses 1.61, 95% CI: 1.37–1.88, assistant nurses 1.25, 95% CI: 1.17–1.34), rather than those in administrative roles (aHR high‐qualified healthcare administrators 1.01 95% CI: 0.76–1.35). Among physicians, the risk was most apparent for psychiatrists (aHR 2.70, 95% CI: 1.21–6.03). For suicide attempts, the risks were primarily observed among registered nurses (aHR 1.22, 95% CI: 1.15–1.29) and assistant nurses (aHR 1.15, 95% CI: 1.12–1.18). Among healthcare workers, assistant nurses had the highest incidence rates for suicide (18.7/100,000 person‐years) and suicide attempts (175.1/100,000 person‐years).

**Conclusions:**

Workers in several healthcare occupations showed a higher risk of suicide relative to non‐healthcare workers with a similar occupational qualification level. Interventions may need to be developed to reduce the risk of suicidal behavior in these groups.

**Summary**

- Significant outcomes
  ○ Healthcare workers in patient care roles, such as physicians, registered nurses, and assistant nurses, had significantly higher suicide risks compared to non‐healthcare workers at a similar occupational qualification level.
  ○ Among physicians, psychiatrists showed the highest suicide risk.
  ○ Assistant nurses had the highest incidence rates for both suicide and suicide attempts among all healthcare occupations studied.
- Limitations
  ○ No full history of participants' working lives before the start of follow‐up due to data unavailability.
  ○ Only capture suicide attempts treated in secondary care, potentially missing less severe cases.
  ○ No information on working conditions between occupations or individuals.

## 1 Introduction

Suicide in healthcare occupations has been studied over a long period, but primarily among physicians. These studies have indicated a higher suicide risk in female physicians [1, 2, 3] and certain specialties, such as general practitioners and psychiatrists [1].

Some studies adjusted for sociodemographic and health indicators to account for potential differences in underlying risks of suicide among different workers [4, 5, 6], while others used a combination of such adjustment and comparison with similar occupational groups [7, 8]. Two Danish studies found a 2–3 times higher risk for physicians [7, 8] when compared to workers in highly qualified occupations, while studies from other countries found a lower magnitude [4, 5, 6] with more uncertainty in the estimates. Similarly, for assistant nurses, one study showed up to an 80% increased risk [6], while another showed no difference in risk [8]. On the other hand, registered nurses were consistently shown to have a higher risk, in the range of 18%–100% [4, 5, 6, 7, 8]. However, there are still limited studies on other occupations within healthcare, for example, dentists [7] or non‐clinical occupations within healthcare [1, 6], for example, medical secretaries and other workers who provide essential administrative support for the healthcare system.

Although stress and working conditions [9] have been cited as reasons for these elevated risks, access to and knowledge of lethal drugs may also contribute to higher rates of suicide in healthcare occupations [7, 8]. Indeed, while case fatality rates for drug poisoning are in general relatively low [10], a higher proportion of drug poisoning as a suicide method has been shown among physicians, registered nurses, dentists, and pharmacists compared to the general population [4, 5, 7]. However, whether this is similar or different for other healthcare occupations, with potentially more limited access to drugs, is unclear.

Moreover, unlike studies on suicides, studies on suicide attempts among healthcare workers have been scarcer. Most previous studies could only estimate prevalence and mainly among physicians [1, 11]. To our knowledge, longitudinal studies in this area are lacking, and most studies [1, 11] relied only on self‐reported information, which might be prone to recall and social desirability bias. Thus, knowledge about the potential effects of work in healthcare occupations on suicide and suicide attempt risk still has important limitations.

### 1.1 Aims of the Study

Using a large, contemporary, register‐based cohort in Sweden, we aimed to address these gaps by determining whether healthcare workers have a higher risk of suicide and/or suicide attempts as compared to similarly qualified workers in non‐healthcare occupations. Moreover, we aimed to determine whether the findings are similar across sexes, across medical specialties, and also to determine the distribution of suicide methods.

## 2 Methods

### 2.1 Study Design and Participants

We identified our study population from the SWIP (Swedish Work, Illness, and labor market Participation) cohort [12], constructed from at least 11 different Swedish national registers, including the Total Population Register (TPR) [13], National Patient Register (NPR), The Longitudinal Integrated Database for Health Insurance and Labour Market Studies (LISA) [14], and Cause of Death Register [15], among others. Information from these registers was linked using unique personal identification numbers assigned to all Swedish residents [16], and delivered in pseudonymized form by Statistics Sweden. The SWIP cohort includes individuals born in 1941–1989 who were residing in Sweden in 2005, which constitutes individuals of working age (16–64 years in 2005), identified through the TPR [13] (*N* = 5,784,437). These individuals were linked to their parents using the Multi‐Generation Register [17]. Information from the register linkage within the SWIP cohort is currently available until December 31, 2020.

**FIGURE 1:**
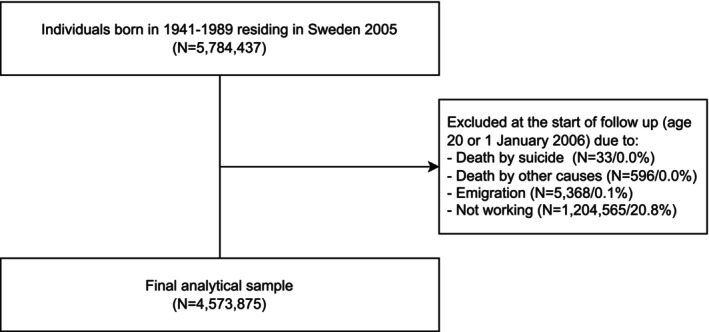
Flowchart of the analytical sample.

For the present study, we started the follow‐up on January 1, 2006 (the year following inclusion in the cohort) or their 20th birthday (when they could reasonably be expected to begin working life), whichever was the latest. Individuals were followed until their first suicide attempt or suicide death (assessed separately), death due to other causes, emigration, 65th birthday, or end of follow‐up on December 31, 2020, whichever came first. We identified their occupational status at baseline using information from LISA [14], and excluded individuals who died or emigrated before baseline of follow‐up (*N* = 5997) [14]. We also excluded individuals who were not working at baseline (e.g., unemployed, students; *N* = 1,204,565). The final cohort consisted of 4,573,875 individuals (Figure 1).

This study was approved by the Regional Ethics Review Board in Stockholm, Sweden (DNR: 2017/1224‐31, 2018/1675‐32, 2022‐02725‐02), which determined that informed consent was not required for the analysis of anonymized register data.

### 2.2 Exposure: Occupations

We identified individuals' occupations at baseline using a combination of the Swedish version of the International Standard Classification of Occupations codes (SSYK96) and Swedish Standard Industrial Classification (SNI2002 and SNI2007) codes (Supporting Information Methods). We further grouped the occupations into high‐ and low‐qualified occupations based on formal educational requirements of the occupations [18] (Table S1).

For physicians, we further identified their specialties using information on the fields of their highest attained education from the LISA database and grouped them into (1) non‐licensed, (2) licensed non‐specialist, (3) specialist in surgery, anesthesia, and intensive care, (4) internal medicine, (5) pediatrics, (6) general practice, (7) psychiatry, (8) other specialists, (9) other medical education, including PhD (Supporting Information Methods).

### 2.3 Outcomes

The suicide outcome was defined as death due to suicide (ICD‐10: X60‐X84, Y10‐Y34), identified from the Cause of Death Register [15]. The suicide attempt outcome was the first recorded suicide attempt, identified from the National Patient Register (NPR) as the first inpatient or outpatient visit during the follow‐up with the ICD codes defined above. Subsequent attempts were not included in the study. We also identified specific methods using the ICD codes (Supporting Information Methods). We included cases with both determined and undetermined intent (~20% of suicides and 50% of suicide attempts), in line with previous Swedish register‐based studies [19, 20] to limit potential underreporting of suicide [21].

### 2.4 Covariates

We identified the following potential confounders: sex (also considered as effect modifier), birth year, and foreign background (defined as having both parents born outside Sweden/not) from the TPR [13]. We also obtained information on factors that might be associated with occupational status [22, 23, 24] and suicidal behaviors [25, 26, 27, 28], such as childhood socioeconomic position, history of mental disorders, somatic disorders, and suicide attempts at baseline. Information on childhood socioeconomic position (SEP) was based on parental occupations obtained from the population and housing censuses [29], measured in the following years: 1960 (for individuals born 1945–1954), 1970 (for individuals born 1955–1964), 1980 (for individuals born 1965–1974), 1990 (for individuals born 1978–1989). This variable was grouped into unskilled manual, skilled manual, assistant non‐manual, intermediate non‐manual, higher non‐manual, farmer, and no registered parental occupation. History of mental and severe somatic disorders diagnoses [30, 31], as well as the history of suicide attempts, were defined as the presence of any ICD‐based diagnoses (Table S2) at any time point up until the start of follow‐up identified from the NPR. For a subset of the population who were born in 1972 onwards, we identified their school grades from the final year of compulsory education (9th grade/age 16) from the School Register held by Statistics Sweden [19] (Supporting Information Methods), which was not available for earlier birth cohorts. We also obtained information on whether the individuals worked within the private/public sector at baseline using information from the LISA database [14].

### 2.5 Statistical Analysis

We summarized the distributions of covariates by exposure and outcome status, respectively, using percentages (%) or mean and standard deviation (SD). We estimated incidence rates of suicide and suicide attempts for each occupation overall and separately by sex. We also presented the distribution of suicide and suicide attempt methods by occupation.

We estimated hazard ratios (HRs) using Cox regression for the risk of suicide and suicide attempts among healthcare workers, with age as the underlying time scale, given the potential age differences across occupational groups at baseline. To enhance the comparability between groups, comparisons were made between specific healthcare occupations and workers in non‐healthcare occupations with similar qualification levels (high‐ and low‐qualified occupations, Table S1), in line with a recent meta‐analysis [3]. Proportional hazard assumptions were assessed using Kaplan–Meier curves and were found not to be violated (Figure S1). Model 1 was adjusted for demographics (sex, birth year, foreign background). Model 2 was adjusted for demographics, socioeconomic status (childhood SEP), and health status at baseline (history of mental disorders, somatic disorders, suicide attempts).

No data was missing on any of the covariates, except in the case of childhood SEP, where 10.0%–18.5% of individuals had no information on childhood SEP. This represents those whose parents had no occupation or were not registered in Sweden and were therefore treated as their own category. Further, individuals with a previous history of suicide attempts were included in the main analysis and accounted for, given that previous suicide attempts are important risk factors for future suicide risk [28] and that excluding these individuals might reduce the power of the analysis, given the rarity of suicide events. We estimated the potential differences between sexes on the risks of suicide and suicide attempts across occupations and tested for interaction using likelihood ratio tests. We repeated similar analyses to estimate potential differences in the risks throughout age at follow‐up and calendar year.

We performed subsequent analyses for physicians by estimating their suicide and suicide attempt risks separately by specialty.

We also performed a supplementary analysis limited to individuals born 1972 onward, adjusting for school grades as a further indicator of baseline differences in risk of suicide.

We performed four sensitivity analyses to minimize the risk for biased conclusions due to potential misclassification of occupational status and suicide attempt outcome by: (1) accounting for the industrial sector, (2) excluding those with a history of suicide attempt, (3) restricting the population to workers over 30, and (4) using a shorter follow‐up period (Supporting Information Methods).

## 3 Results

4,573,875 individuals were followed over 55,880,902.8 person‐years. Healthcare workers had a higher proportion of women, a history of mental and somatic disorders, and a history of suicide attempts, as compared to other workers (Table 1). Among healthcare workers in high‐qualified occupations, registered nurses made up the largest occupation (43.8%), followed by healthcare administrators, therapists, other allied health professionals, and physicians (Table S3). Healthcare workers in low‐qualified occupations were predominantly assistant nurses (96.2%, Table S3).

**TABLE 1:** Demographic, health, and socioeconomic characteristics of the study population (*N* = 4,573,875), based on occupations.

Variables	Categories	High‐qualified		Low‐qualified	
		Healthcare workers	Non‐healthcare workers	Healthcare workers	Non‐healthcare workers
		*N* = 243,183	*N* = 1,789,076	*N* = 514,726	*N* = 2,026,890
		*n* (%)	*n* (%)	*n* (%)	*n* (%)
Follow‐up time (years)	Mean (SD)	11.9 (4.5)	11.9 (4.6)	12.5 (4.2)	12.5 (4.2)
	Median (IQR)	15.0 (8.9–15.0)	15.0 (8.8–15.0)	15.0 (11.4–15.0)	15.0 (11.5–15.0)
Sex	Female	197,708 (81.3)	878,252 (49.1)	442,863 (86.0)	773,643 (38.2)
	Male	45,475 (18.7)	910,824 (50.9)	71,863 (14.0)	1,253,247 (61.8)
Birth year	1941–1950	63,023 (25.9)	450,233 (25.2)	95,911 (18.6)	368,000 (18.2)
	1951–1960	73,813 (30.4)	429,772 (24.0)	114,479 (22.2)	383,414 (18.9)
	1961–1970	60,410 (24.8)	488,233 (27.3)	116,009 (22.5)	452,783 (22.3)
	1971–1980	42,260 (17.4)	376,982 (21.1)	103,092 (20.0)	463,814 (22.9)
	1981–1989	3677 (1.5)	43,856 (2.5)	85,235 (16.6)	358,879 (17.7)
Age at baseline	Mean (SD)	46.3 (10.8)	45.0 (11.4)	40.9 (13.3)	39.9 (13.4)
Foreign background	No	211,678 (87.0)	1,610,514 (90.0)	424,190 (82.4)	1,690,060 (83.4)
	Yes	31,505 (13.0)	178,562 (10.0)	90,536 (17.6)	336,830 (16.6)
History of mental disorders	No	228,531 (94.0)	1,697,288 (94.9)	461,228 (89.6)	1,865,434 (92.0)
	Yes	14,652 (6.0)	91,788 (5.1)	53,498 (10.4)	161,456 (8.0)
History of somatic disorders	No	215,371 (88.6)	1,605,858 (89.8)	456,794 (88.8)	1,822,806 (89.9)
	Yes	27,812 (11.4)	183,218 (10.2)	57,932 (11.3)	204,084 (10.1)
History of suicide attempts	No	239,463 (98.5)	1,767,849 (98.8)	498,526 (96.9)	1,983,219 (97.9)
	Yes	3720 (1.5)	21,227 (1.2)	16,200 (3.2)	43,671 (2.2)
Parental socioeconomic positions in childhood	Unskilled manual	43,042 (17.7)	330,686 (18.5)	139,364 (27.1)	541,699 (26.7)
	Skilled manual	47,606 (19.6)	367,507 (20.5)	128,107 (24.9)	511,137 (25.2)
	Assistant non‐manual	27,300 (11.2)	234,285 (13.1)	40,693 (7.9)	177,172 (8.7)
	Intermediate non‐manual	52,225 (21.5)	422,345 (23.6)	63,966 (12.4)	267,124 (13.2)
	Higher non‐manual	27,049 (11.1)	172,228 (9.6)	19,773 (3.8)	77,246 (3.8)
	Farmer	14,647 (6.0)	83,013 (4.6)	27,569 (5.4)	113,162 (5.6)
	No registered occupation	31,314 (12.9)	179,012 (10.0)	95,254 (18.5)	339,350 (16.7)

Those who died by or attempted suicide were more likely to have a history of mental and somatic disorders, as well as a history of suicide attempts (Table S4).

### 3.1 Suicide

Among workers in high‐qualified occupations, psychologists, psychotherapists, counselors (hazard ratio [HR] 1.62, 95% CI: 1.06–2.46), registered nurses (HR 1.61, 95% CI: 1.37–1.88), physicians (HR 1.57, 95% CI: 1.23–2.00), and therapists and other allied health professionals (HR 1.48, 95% CI: 1.12–1.96) showed higher risks of suicide (Table 2). Among low‐qualified healthcare occupations, assistant nurses (HR 1.25, 95% CI: 1.17–1.34) showed a higher risk of suicide, while low‐qualified healthcare administrators showed a lower risk (HR 0.71, 95% CI: 0.49–1.04, Table 2).

**TABLE 2:** Number of workers, number of cases, incidence rates (95% CI), and hazard ratios (95% CI) for suicide and suicide attempt by occupations.

Occupational qualification level	Occupations	Suicide				Suicide attempt			
		*N* (*n*)	Incidence rate per 100,000 person‐years (95% CI)	HR (95% CI)		*N* (*n*)	Incidence rate per 100,000 person‐years (95% CI)	HR (95% CI)	
				Model 1^a^	Model 2^b^			Model 1^a^	Model 2^b^
High‐qualified									
	Physicians	32,404 (69)	18.4 (14.6–23.3)	1.49 (1.17–1.90)	1.57 (1.23–2.00)	32,404 (259)	69.6 (61.6–78.6)	0.89 (0.79–1.01)	0.94 (0.83–1.06)
	Registered nurses	106,600 (177)	13.8 (11.9–15.9)	1.76 (1.50–2.06)	1.61 (1.37–1.88)	106,600 (1248)	97.8 (92.5–103.3)	1.29 (1.22–1.37)	1.22 (1.15–1.29)
	Dentists and dental hygienists	9077 (11)	10.5 (5.8–19.0)	1.07 (0.59–1.93)	1.14 (0.63–2.07)	9077 (80)	76.8 (61.7–95.7)	0.99 (0.79–1.23)	1.04 (0.84–1.30)
	Psychologists, psychotherapists, counselors	11,848 (22)	17.0 (11.2–25.9)	1.81 (1.19–2.76)	1.62 (1.06–2.46)	11,848 (106)	82.5 (68.2–99.8)	1.08 (0.89–1.30)	1.02 (0.84–1.24)
	Pharmacists and prescriptionists	6297 (7)	10.5 (5.0–22.1)	1.37 (0.65–2.88)	1.37 (0.65–2.88)	6297 (53)	80.3 (61.3–105.1)	1.03 (0.79–1.36)	1.06 (0.81–1.39)
	Therapists and other allied health professionals	33,730 (51)	12.1 (9.2–16.0)	1.48 (1.12–1.96)	1.48 (1.12–1.96)	33,730 (338)	80.9 (72.7–90.0)	1.07 (0.96–1.19)	1.09 (0.98–1.22)
	Healthcare administrators (high‐qualified)	43,227 (48)	9.6 (7.2–12.7)	1.10 (0.83–1.47)	1.01 (0.76–1.35)	43,227 (474)	95.2 (87.0–104.2)	1.23 (1.12–1.35)	1.16 (1.05–1.27)
	Non‐healthcare workers (high‐qualified)	1,789,076 (2379)	11.2 (10.8–11.7)	1	1	1,789,076 (16,134)	76.4 (75.3–77.6)	1	1
Low‐qualified									
	Assistant nurses	495,130 (1160)	18.7 (17.7–19.8)	1.44 (1.34–1.55)	1.25 (1.17–1.34)	495,130 (10,691)	175.1 (171.9–178.5)	1.27 (1.24–1.30)	1.15 (1.12–1.18)
	Healthcare administrators (low‐qualified)	19,596 (27)	11.4 (7.8–16.6)	0.72 (0.49–1.05)	0.71 (0.49–1.04)	19,596 (266)	113.3 (100.5–127.8)	0.81 (0.72–0.91)	0.82 (0.72–0.92)
	Non‐healthcare workers (low‐qualified)	2,026,890 (5427)	21.4 (20.9–22.0)	1	1	2,026,890 (37,520)	150.0 (148.5–151.5)	1	1

^a^ Adjusted for sex, birth year, and foreign background.

^b^ Adjusted for sex, birth year, foreign background, parental socioeconomic positions, history of mental disorders, history of somatic disorders, history of suicide attempts.

**FIGURE 2:**
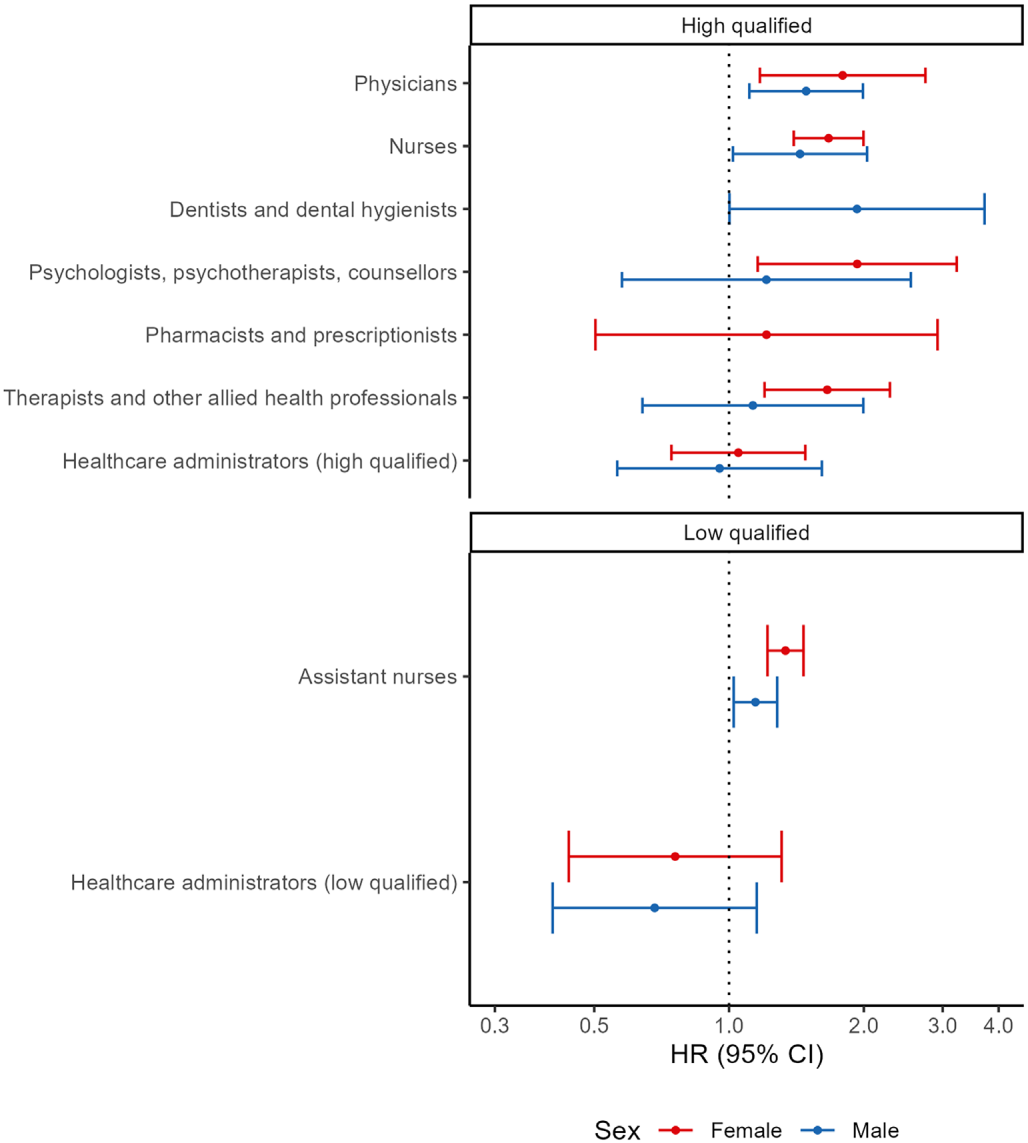
Hazard ratios (95% CI) for suicide by occupations stratified by sex. Adjusted by birth year, foreign background, parental socioeconomic positions, history of mental disorders, history of somatic disorders, history of suicide attempts. Estimates with a low number of observations were not presented. *p*‐values for interaction between occupations and sex are 0.24 (higher qualified) and 0.11 (lower qualified).

The increased risks of suicide in physicians, registered nurses, and assistant nurses were seen in both women and men (Figure 2, Table S13). While the likelihood ratio tests did not show statistically significant interaction effects for sex, we observed higher HRs for women (Figure 2, Table S13). Nevertheless, it should be noted that the absolute difference (based on the difference in incidence rates) was mostly higher among men. For example, male physicians had an additional 8.1 suicide cases per 100,000 person‐years compared to other male high‐qualified non‐healthcare workers, whereas female physicians had an additional 5.7 suicide cases per 100,000 person‐years compared to other female high‐qualified non‐healthcare workers. For psychologists, psychotherapists, counselors, and therapists, and other allied health professionals, the certainty of the estimates was more apparent among women than among men.

A higher proportion of healthcare workers who died by suicide died due to drug poisoning (Table S5). Other workers had a higher proportion of deaths due to drowning and suffocation.

Suicide rates among different physician specialties can be found in Table S7. Among different physician specialties, we found that the increased risk of suicide among physicians was stronger for psychiatrists (HR 2.70, 95% CI: 1.21–6.03), general practitioners (HR 2.16, 95% CI 1.30–3.60), and specialists within surgery, anesthesia, and intensive care (HR 2.46, 95% CI 1.48–4.09, Table 3).

**TABLE 3:** Hazard ratios (95% CI) for suicide and suicide attempt among physicians based on their specialties.

Occupations^a^	Suicide		Suicide attempt	
	HR (95% CI)		HR (95% CI)	
	Model 1^b^	Model 2^c^	Model 1^b^	Model 2^c^
Physicians				
Non‐licensed physicians	0.99 (0.52–1.92)	0.97 (0.50–1.87)	0.86 (0.67–1.11)	0.87 (0.68–1.12)
Licensed non‐specialist physicians	‐^d^	‐^d^	0.86 (0.48–1.56)	0.93 (0.51–1.68)
Surgery, anesthesia, intensive care	2.16 (1.30–3.60)	2.46 (1.48–4.09)	1.15 (0.86–1.55)	1.28 (0.95–1.72)
Internal medicine	1.36 (0.56–3.27)	1.44 (0.60–3.47)	0.47 (0.25–0.88)	0.49 (0.27–0.92)
Pediatrics	‐^d^	‐^d^	‐^d^	‐^d^
General practice	2.25 (1.35–3.74)	2.16 (1.30–3.60)	1.14 (0.85–1.53)	1.13 (0.84–1.53)
Psychiatry	3.22 (1.45–7.19)	2.70 (1.21–6.03)	1.22 (0.72–2.05)	1.11 (0.66–1.88)
Radiology, clinical laboratory, other specialties	1.27 (0.53–3.06)	1.34 (0.56–3.22)	0.75 (0.47–1.21)	0.80 (0.50–1.29)
Other medical education, including PhD	1.05 (0.55–2.03)	1.22 (0.63–2.35)	0.89 (0.66–1.20)	1.02 (0.75–1.37)
Unknown	‐^d^	‐^d^	0.60 (0.33–1.08)	0.66 (0.37–1.20)
Non‐healthcare workers (high‐qualified)	1	1	1	1

^a^ Other healthcare workers were grouped in separate categories in the model. Data not presented.

^b^ Adjusted for sex, birth year, foreign background.

^c^ Adjusted for sex, birth year, foreign background, parental socioeconomic positions, history of mental disorders, history of somatic disorders, history of suicide attempts.

^d^ Estimates were not presented due to a low number of observations.

### 3.2 Suicide Attempts

Among healthcare workers in high‐qualified occupations, only registered nurses (HR 1.22, 95% CI: 1.15–1.29) and healthcare administrators (HR 1.16, 95% CI: 1.05–1.27) showed increased risks of suicide attempts (Table 2). Among healthcare workers in low‐qualified occupations, assistant nurses (HR 1.15, 95% CI: 1.12–1.18) showed an increased risk of suicide attempts, while low‐qualified healthcare administrators showed a decreased risk (HR 0.82, 95% CI: 0.72–0.92, Table 2).

**FIGURE 3:**
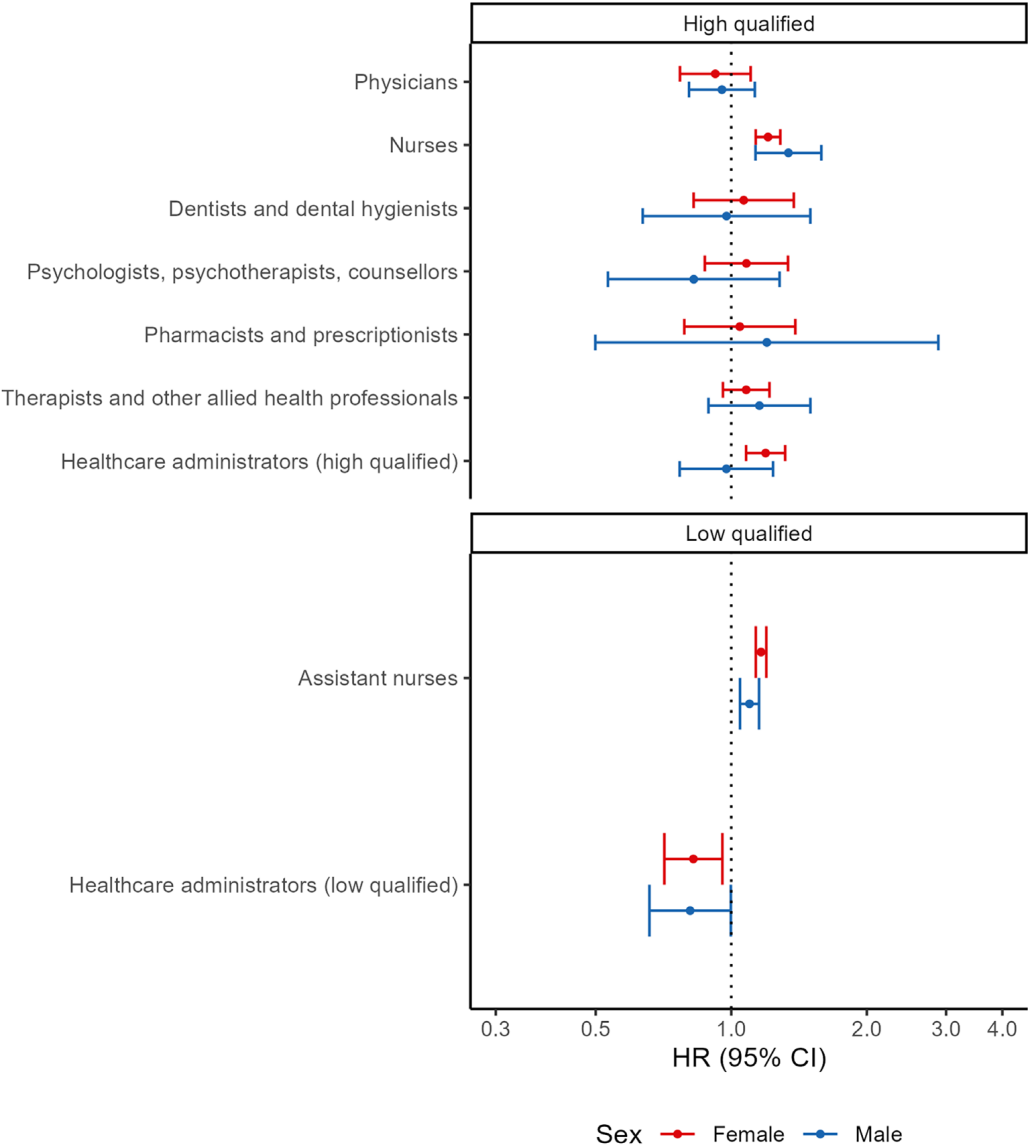
Hazard ratios (95% CI) for suicide attempt by occupations stratified by sex. Adjusted by birth year, foreign background, parental socioeconomic positions, history of mental disorders, history of somatic disorders, history of suicide attempts. *p*‐values for interaction between occupations and sex are 0.60 (higher qualified) and 0.11 (lower qualified).

The increased risks of suicide attempts in registered nurses and assistant nurses were seen in both sexes (Figure 3, Table S14). The decreased risk in lower‐qualified healthcare administrators was seen most clearly in women. We did not observe statistically significant effects of interaction by sex through the likelihood ratio tests. However, we observed that the higher risk of suicide attempt among high‐qualified healthcare administrators was limited to women.

Registered nurses and high‐qualified healthcare administrators showed a higher proportion of suicide attempts due to drug poisoning, as compared to other high‐qualified workers (Table S6). Assistant nurses and low‐qualified healthcare administrators showed similar patterns, compared to other workers in low‐qualified occupations.

The risks for suicide attempts were not as elevated across different physicians specialties (Table 3).

### 3.3 Supplementary and Sensitivity Analyses

The risks of suicide and suicide attempts were similar regardless of age at follow‐up (Figures S2, S3) or calendar year (Figures S4, S5) for most healthcare workers. The risks were also similar to the main results when we additionally adjusted for school grades among individuals born in 1972 and onward (Table S8).

The results did not deviate from those in the main analysis when we made restrictions to workers within the public sector (Table S9). When including only individuals without a previous history of suicide attempts, we observed slightly higher HRs (Table S10). Additionally, the results did not change when we modified the start of follow‐up to include individuals at least 30 years of age only (Table S11). Moreover, the results were largely similar when we included shorter follow‐up times (Table S12).

## 4 Discussion

### 4.1 Key Findings

To our knowledge, this is the largest and most comprehensive cohort study to date examining healthcare occupations and the risks of suicide and suicide attempts. After controlling for a range of sociodemographic and health indicators, physicians, registered nurses, assistant nurses, psychologists, psychotherapists, counselors, therapists, and other allied health professionals were found to have a 25%–60% higher risk of suicide in comparison with non‐healthcare workers with similar occupational qualification levels. However, increased risks of suicide attempts were found in fewer healthcare occupations: registered nurses, assistant nurses, and highly qualified healthcare administrators. While increased risks for suicide were seen in both women and men, we often observed higher and more certain risks in women. Nevertheless, the absolute difference in suicide rates was higher among men. Among physicians, the increased risk of suicide was apparent among specialists within psychiatry, surgery, anesthesia, intensive care, and general practice. Additionally, drug poisoning was found to be a more common method of suicide and suicide attempts in, for instance, registered nurses and physicians.

### 4.2 Strengths and Limitations

In this study, we included virtually the whole Swedish working population in 2006, followed for up to 15 years, enabling us to retain sufficient power to assess the risk of relatively rare outcomes like suicide. We were also able to ascertain suicide attempt outcomes over time based on clinical diagnosis across healthcare and non‐healthcare occupations, something that has been lacking previously. Additionally, we included broader groups of occupations, including both clinical and non‐clinical occupations within healthcare, which have only been assessed to a limited extent. By using high‐quality national registers, we were able to avoid a loss to follow‐up. The Swedish registers have previously been shown to have high validity and completeness for both occupations [14] and suicide or suicide attempts [15, 32]. Further, control over potential confounding was comprehensive. For individuals born in 1972 and later, we were even able to account for differences in grade‐point average (GPA) in school, which is predictive of later risk of suicide attempt [19].

Nevertheless, this study has some limitations. Due to the nature of the data, we were only able to follow the participants from a fixed start of follow‐up (i.e., January 2006) and did not have a full history of their working lives. We could only capture cases of suicide attempts severe enough to be treated within secondary care. Further, we only used information on occupation at baseline, which might lead to over‐ or underestimation of the estimated risks due to post‐baseline changes in occupations. However, we performed another analysis with a shorter follow‐up time to minimize such misclassifications, and the results were largely similar to the main analysis. Additionally, for physicians, we only had information on their medical specialties based on the fields of their highest education, which might lead to physicians with double degrees (e.g., MD‐PhD) being grouped into “other medical education, including PhD” instead of their clinical specialties. This could potentially lead to underestimation of effects for clinical specialties. Moreover, we did not have any direct information on differences in psychosocial working conditions between occupations or individuals. Also, missing information on factors that are associated with both occupational choices and suicide behaviors, such as personality, might have led to residual confounding in the observed associations.

### 4.3 Comparison With Previous Studies

The increased risk of suicide deaths found among female and male physicians, as compared to workers in other high‐qualified occupations, is consistent with two earlier studies using Danish data [7, 8], although the effect size were smaller in the present study. Like our study, the estimates in these studies were adjusted for a range of covariates combined with a matched comparison group (i.e., teachers, which is a high‐qualified occupation). On the other hand, a US study [6] did not find an increased risk of suicide among physicians. In that study [6], however, physicians were compared with all other workers, both in high‐ and low‐qualified occupations.

Considerably more studies were directed toward estimating standardized suicide rates between physicians and other workers [1, 2, 3]. The most recent meta‐analysis, including 39 studies spanning from 1935 to 2020, showed a 76% higher rate in female physicians but only a 5% higher rate for male physicians, with more uncertainty in the estimate [3]. However, when this study [3] compared male physicians to other professional groups with similar socioeconomic status, it also found an increase in rate ratio, like our study. Our findings were also in line with a recent Norwegian study, which found higher rates of suicide among physicians compared to others with higher education [33].

### 4.4 Potential Explanations

Increased suicide risk among healthcare workers, and especially those involved in patient care, suggests that factors linked to specific occupations are of particular importance. Mental health problems caused by exposure to human suffering and death have been discussed, although this is more uncertain in relation to healthcare workers' everyday experiences [34]. Furthermore, there are indications that, for example, veterinarians' work with euthanasia is associated with a higher likelihood of having serious suicidal thoughts [35]. Increased risk among healthcare workers involved in patient care may also be facilitated by knowledge and access to lethal means, as indicated by the higher proportion of drug poisoning as the method of suicide in this group. In our study, certain occupations, such as physicians, were shown to have an increased risk of suicide but not of suicide attempts. This may indicate that workers in such occupations have a higher capacity to make fatal attempts resulting in more suicide deaths.

In a broader context, mental disorders have been acknowledged as a major predictor of suicide [36]. However, studies on the risks of common mental disorders in healthcare workers have shown both higher [37] and lower [38] risks. While we have accounted for the workers' history of mental disorders at baseline, our study was not aimed at investigating to what extent mental health problems during follow‐up contribute to the increased risks of suicide and suicide attempts. At the same time, due to the nature of the available data, some cases of previous mental health diagnoses, which were adjusted for, may have occurred after the person was established in their occupation, leading to a potential over‐adjustment. Thus, the estimates presented can be seen as more conservative relative risks.

There may be characteristics or personality traits that, to some extent, correlate with the choice of occupation and with the risk of suicide, for example, academic performance, which has been shown to predict later suicidal behaviors [19]. In our study, controlling for academic performance did not meaningfully change the associations. Nevertheless, there are other relevant characteristics of individuals that we could not measure. For example, Kendler et al. [39] showed that within the Swedish population, specialist physicians, psychologists, psychotherapists, and psychiatric nurses have increased family‐genetic risk for depression and anxiety. Consequently, this might later contribute to their increased risk of suicidal behavior. In line with this, a recent Swedish study found that university students in nursing/healthcare had an increased suicide risk, partly attributable to a history of hospitalization due to mental disorder or self‐harm [40].

Psychosocial workplace risk factors such as long working hours, low job control, and harassment [41] may also contribute to explaining the risks of suicide attempts and suicide observed in this study. A Swedish study showed that a higher proportion among registered nurses and assistant nurses report low job control, as compared to other workers [42]. In turn, low control at work is associated with suicide and suicide attempts as well as depression in the working population in Sweden [12, 20]. Moreover, there is evidence that harassment at work may be associated with an increased risk of suicide among workers in Sweden [31]. However, it is currently not known whether harassment is more common among healthcare workers than among others.

### 4.5 Implications

Healthcare occupations are relatively standardized across settings. However, there might be differences in how the healthcare system is organized. Therefore, our findings might be best generalized to settings with similar healthcare systems and general working conditions.

Future studies are needed to disentangle individual and workplace‐level factors as explanations for the increased suicide risk in occupations indicated by the present study and previous ones.

As noted in systematic reviews, there is a lack of intervention programs for suicide prevention among healthcare workers [43, 44]. Still, these reviews identified two intervention studies for suicidal ideation among junior physicians showing potential benefits of cognitive behavioral therapy [45] but not yoga or group fitness [46]. However, studies on other healthcare workers and on broader suicide‐related outcomes, such as suicide attempts, are still needed.

Some aspects, like the higher availability of lethal drugs among physicians, registered nurses, and some other occupations, may be difficult to target. On the other hand, given that mental disorders might play a role in the risk of suicidal behavior, workplace mental health programs might be beneficial for suicide prevention, even if more evaluation has been called for [9]. Previous studies have indicated that physician‐directed intervention might be beneficial in reducing common mental disorder symptoms [44]. Future studies should evaluate to what extent this might also be beneficial in reducing suicidal behaviors, also in other healthcare workers. Interventions targeting specific psychosocial working conditions are another possibility, although their impact on workers' risk of suicide remains to be studied [9].

## 5 Conclusions

Workers in several healthcare occupations, for example, physicians, registered nurses, and assistant nurses, had a higher risk of suicide when compared with non‐healthcare workers with a similar occupational qualification level. Increased risk of suicide attempts was seen among registered nurses and assistant nurses. Our study provides an understanding of patterns and elevated risks within certain occupations at the population level. Nevertheless, it should be acknowledged that suicidal behavior is complex and may be influenced by multiple factors. Therefore, future research should aim to uncover precise mechanisms behind these associations and potential for prevention efforts in these populations.

## Contributors

A.N. conceived the study and wrote the protocol. D.F., E.B., K.Y.P., M.A., and T.B. contributed to the protocol. AN extracted the data and performed the data analysis. A.N., D.F., E.B., K.Y.P., M.A., T.B., K.K., and T.H. contributed to data interpretation. A.N. and D.F. wrote the initial draft, and all authors contributed to writing the final manuscript. A.N. and D.F. are the guarantors of the study.

## Ethics Statement

The authors assert that all procedures contributing to this work comply with the ethical standards of the relevant national and institutional committees on human experimentation and with the Helsinki Declaration of 1975, as revised in 2008.

## Conflicts of Interest

The authors declare no conflicts of interest.

## Peer Review

The peer review history for this article is available at https://www.webofscience.com/api/gateway/wos/peer‐review/10.1111/acps.70018.

## Supporting information


**Table S1.** Classification of healthcare occupations based on the Swedish Standard Classification of Occupations (SSYK) and the Swedish Standard Industrial Classification (SNI).
**Table S2.** ICD‐codes for mental and severe somatic disorders diagnoses.
**Table S3.** Distribution of healthcare workers based on occupational qualification level (*N* = 757,909).
**Table S4.** Demographic, health, and socioeconomic characteristics of the study population (*N* = 4,573,875), based on suicide and suicide attempt outcomes for each occupational qualification level.
**Table S5.** Distribution of suicide methods by occupations (*N* = 9378).
**Table S6.** Distribution of first suicide attempt methods by occupations (*N* = 67,169).
**Table S7.** Incidence rates of suicide and suicide attempt among physicians based on their specialties.
**Table S8.** Hazard ratios (95% CI) of suicide and suicide attempt among individuals born from 1972 onwards.
**Table S9.** Hazard ratios (95% CI) for suicide and suicide attempt among individuals working within public sectors.
**Table S10.** Number of cases, follow‐up time, incidence rates (95% CI), and hazard ratios (95% CI) for suicide attempt by occupations, among individuals with no previous history of suicide attempts.
**Table S11.** Hazard ratios (95% CI) for suicide and suicide attempt with later start of follow‐up (30th birthday or 1 January 2006).
**Table S12.** Hazard ratios (95% CI) for suicide and suicide attempt with earlier end of follow‐up (31 December 2010).
**Table S13.** Number of workers, number of cases, incidence rates (95% CI), and hazard ratios (95% CI) for suicide across occupations stratified by sex.
**Table S14.** Number of workers, number of cases, incidence rates (95% CI), and hazard ratios (95% CI) for suicide attempt across occupations stratified by sex.
**Figure S1.** Kaplan–Meier curves for: (A) suicide and (B) suicide attempt among workers in [1] high qualified and [2] low‐qualified occupations.
**Figure S2.** Hazard ratios (95% CI) for suicide by occupations stratified by age at follow up. Adjusted for birth year, foreign background, parental socioeconomic positions, history of mental disorders, history of somatic disorders, history of suicide attempts. Estimates with low number of observations were not presented. *p*‐values for interaction between occupations and age are 0.55 (higher qualified) and 0.22 (lower qualified).
**Figure S3.** Hazard ratios (95% CI) for suicide attempt by occupations stratified by age at follow up. Adjusted for birth year, foreign background, parental socioeconomic positions, history of mental disorders, history of somatic disorders, history of suicide attempts. Estimates with low number of observations were not presented. *p*‐values for interaction between occupations and age are 0.10 (higher qualified) and 0.08 (lower qualified).
**Figure S4.** Hazard ratios (95% CI) for suicide by occupations stratified by calendar year. Adjusted for birth year, foreign background, parental socioeconomic positions, history of mental disorders, history of somatic disorders, history of suicide attempts. Estimates with low number of observations were not presented. *p*‐values for interaction between occupations and age are 0.30 (higher qualified) and 0.00 (lower qualified).
**Figure S5.** Hazard ratios (95% CI) for suicide attempt by occupations stratified by calendar year. Adjusted for birth year, foreign background, parental socioeconomic positions, history of mental disorders, history of somatic disorders, history of suicide attempts. Estimates with low number of observations were not presented. *p*‐values for interaction between occupations and age are 0.02 (higher qualified) and 0.07 (lower qualified).

## Data Availability

The data that support the findings of this study are based on information from the national Swedish registers available from Statistics Sweden (SCB.se). As third‐party holders, we do not have the rights to share the data publicly. More information is available from Statistics Sweden or the corresponding author. Original study protocol can be accessed from https://doi.org/10.17605/OSF.IO/AJB7H.
