# Supplementary material for: Healthcare Occupations, Suicides, and Suicide Attempts: A Cohort Study Based on the Working Population in Sweden
Source: Acta Psychiatr Scand. 2025 Jul 20;152(5):360–71. doi: 10.1111/acps.70018 (PMC12488363; doi:10.1111/acps.70018)
Supplement: Supplementary file 1 — Table S1. Classification of healthcare occupations based on the Swedish Standard Classification of Occupations (SSYK) and the Swedish Standard Industrial Classification (SNI). Table S2. ICD‐codes for mental and severe somatic disorders diagnoses. Table S3. Distribution of healthcare workers based on occupational qualification level (N = 757,909). Table S4. Demographic, health, and socioeconomic characteristics of the study population (N = 4,573,875), based on suicide and suicide attempt outcomes for each occupational qualification level. Table S5. Distribution of suicide methods by occupations (N = 9378). Table S6. Distribution of first suicide attempt methods by occupations (N = 67,169). Table S7. Incidence rates of suicide and suicide attempt among physicians based on their specialties. Table S8. Hazard ratios (95% CI) of suicide and suicide attempt among individuals born from 1972 onwards. Table S9. Hazard ratios (95% CI) for suicide and suicide attempt among individuals working within public sectors. Table S10. Number of cases, follow‐up time, incidence rates (95% CI), and hazard ratios (95% CI) for suicide attempt by occupations, among individuals with no previous history of suicide attempts. Table S11. Hazard ratios (95% CI) for suicide and suicide attempt with later start of follow‐up (30th birthday or 1 January 2006). Table S12. Hazard ratios (95% CI) for suicide and suicide attempt with earlier end of follow‐up (31 December 2010). Table S13. Number of workers, number of cases, incidence rates (95% CI), and hazard ratios (95% CI) for suicide across occupations stratified by sex. Table S14. Number of workers, number of cases, incidence rates (95% CI), and hazard ratios (95% CI) for suicide attempt across occupations stratified by sex. Figure S1. Kaplan–Meier curves for: (A) suicide and (B) suicide attempt among workers in [1] high qualified and [2] low‐qualified occupations. Figure S2. Hazard ratios (95% CI) for suicide by occupations stratified by age at fol [file ACPS-152-360-s001.pdf]

Supplementary files
Healthcare occupations, suicides, and suicide attempts: A cohort study based on the working population in Sweden

Alicia Nevriana, Emma Brulin, Tomas Hemmingsson, Melody Almroth, Kuan-Yu Pan, Theo Bodin, Katarina Kjellberg, Daniel Falkstedt

Supplementary methods

Exposure: Healthcare occupations

The occupations were primarily identified based on the Swedish version of the International Standard Classification of Occupations codes (*Standard för svensk yrkesklassificering 1996/*SSYK96) at a 4-digit level in the year before the start of follow-up since information on occupations was only available on a yearly basis (1). Where 4-digit codes were not available, 3-digit codes were used to classify an individual's occupation. We also identified the workplace sector at baseline (healthcare/not) using Swedish Standard Industrial Classification (*Standard för svensk näringsgrensindelning*/SNI version 2002 and 2007) codes. For certain occupations where the category might include non-healthcare-specific occupations (e.g., counsellors), we only included individuals who were also identified as working within healthcare sectors based on the SNI codes. We also identified individuals with occupational codes not listed in Supplementary Table 1 but with SNI codes indicating healthcare sectors as 'healthcare administrators.'

In the sensitivity analysis where the start date of follow-up might include 1 January 2006 or 30th birthday (whichever was the latest), we also used SSYK version 2012 (SSYK2012) to identify the occupations for individuals who started their follow-up in or after 2015.

For physicians, the following codes are used to identify the physicians' specialties based on the information from the fields of highest attained education (SUN2000Inr) in the LISA database: 1) non-licensed (721a), 2) licensed non-specialist (721b), 3) specialist in surgery, anaesthesia, and intensive care (721c), 4) internal medicine (721d, 721f), 5) paediatrics (721e), 6) general practice (721g), 7) psychiatry (721h), 8) other specialists (721i, 721j, 721k), 9) other medical education, including PhD (721x).

Outcome: Suicide deaths and attempts

The following methods were identified: poisoning due to drugs (ICD-10: X60-X64, Y10-Y14), poisoning due to other substances (ICD-10: X65-X69, Y15-Y19), suffocation and drowning, including hanging (ICD-10: X70-X71, Y20-Y21), firearms (ICD-10: X72-X75, Y22-Y25), burns (ICD-10: X76-X77, Y26-Y27), cutting (ICD-10: X78, Y28), and others (ICD-10: X79-X84, Y29-Y34).

Covariates

For individuals born from 1972 who graduated in 1988-1997, we used information on the Grade Point Average (GPA) available within the School Register (2) and categorized it into quintiles according to the year of graduation. Afterwards, due to changes in the grading system, for individuals who graduated 1998-onwards, information on the summary school grades (3) was used and categorized into quintiles according to year of graduation.

Sensitivity analyses

We performed four sensitivity analyses. First, we only included healthcare workers who were working within the public sector, to reduce potential misclassifications, since it was previously discovered that some healthcare workers within the private sector (primarily registered nurses) were misclassified into other healthcare professions (4). Second, for suicide attempts, we excluded individuals with a history of suicide attempts to only capture first-time suicide attempt and ensure that the exposure preceded the outcome (5). Third, we modified the start of follow-up to include individuals either on their 30th birthday or 1 January 2006, whichever was the latest, to minimize potential exposure misclassification among the youngest group. Fourth, given that individuals' occupations might change during the long follow-up time, we shortened the follow-up (to 31 December 2010), to minimize the potential influence of misclassification of occupations.

Supplementary tables

Table S1 Classification of healthcare occupations based on the Swedish Standard Classification of Occupations (SSYK) and the Swedish Standard Industrial Classification (SNI)

Occupational qualification level	Occupations	Descriptions	SSYK96[a]	SSYK2012[b]	SNI2002[c]	SNI2007[d]
High-qualified	Physicians	Physicians	2221	2211-2219		
	Registered nurses	Registered nurses and midwives	2231-2236, 3231-3239	2221-2239		
	Dentists and dental hygienists	Dentists	2222	2260		
		Dental hygienists	3225	3250		
	Psychologists, psychotherapists, counselors	Psychologists and psychotherapists	2491	2241, 2242		
		Counselors	2492	2662	851	861-869
	Pharmacists and prescriptionists	Pharmacists	2224	2281		
		Prescriptionists	3228	3213		
	Therapists and other allied health professionals	Occupational therapists	3221	2273		
		Physiotherapists	3226	2271, 2272		
		Speech therapists, audiologists, and other therapists	2225, 3229	2283, 2289	851	861-869
		Dieticians	3223	2282		
		Opticians	3224	2284		
		Medical technicians	3134, 3240	3211, 3212, 3214	851	861-869
		Medical physicist	2111	2111	851	861-869
	Healthcare administrators (high-qualified)	Medical secretaries	4112	4117	851	861-869
		Other workers providing administrative support within the healthcare	0, 1, 2, 3 (except 3462), 411, 412, 4132, 414, 419, 4214, 4215	0, 1, 2, 3 (except 3451), 411, 4212, 4132, 422, 4410, 4430, 5111, 5113, 5242	851	861-869
	Non-healthcare workers (high-qualified)	Other occupations not included above	0 (military occupations), 1 (legislators, senior officials and managers, 2 (professionals), 3 (technicians and associate professionals, except 3462), 411, 412, 4132, 414, 419, 4214, 4216	0, 1, 2, 3 (except 3451), 411, 4212, 4132, 422, 4410, 4430, 5111, 5113, 5242		
Low qualified	Assistant nurses	Assistant nurses	5132-5134	5321-5326, 5330, 5341-5343		
		Dental nurses	5135	5350		
		Other healthcare workers providing patient care	2229, 5139	2131, 5349	851	861-869
	Healthcare administrators (low-qualified)	Other workers providing administrative support within the healthcare	Rest of codes	Rest of codes	851	861-869
	Non-healthcare workers (low-qualified)	Other occupations not included above	Rest of codes (e.g., 6 (agricultural workers), 7 (construction workers), 8 (transport workers), 9 (elementary occupations)	Rest of codes		

[a]Applicable up to 2013
[b]Applicable 2014 onwards
[c]Applicable up to 2006
[d]Applicable 2007 onwards

Table S2 ICD-codes for mental and severe somatic disorders diagnoses

Diagnosis		ICD-6	ICD-7	ICD-8	ICD-9	ICD-10
Mental disorders		300-326	300-326	290-315	290-319	F00-F99
Somatic disorders	Myocardial infarction	420,1	420,1	410; 411; 412,01; 412,91	410; 412	I21; I22; I25.2
	Congestive heart failure	422,2; 434,1; 434,2	422,21; 422,22; 434,1; 434,2	425,08; 425,09; 427,0; 427,1; 428	402; 404; 425E; 425F; 425H; 425W; 425X; 428	I11.0; I13.0; I13.2; I25.5; I42.0; I42.6; I42.7; I42.8; I42.9; I43; I50
	Peripheral vascular disease	450; 451; 453	450; 451; 453	440; 441; 443,1; 443,9	440; 441; 443B; 443X; 447B; 557	I70; I71; I73.1; I73.8; I73.9; I77.1; I79.0; I79.2; K55
	Cerebrovascular disease	330-334	330-334	430-438	430-438	G45; I60-I64; I67; I69
	Chronic obstructive pulmonary disease (COPD)	502; 527,1	502; 527,1	491; 492	491; 492; 496	J43; J44
	Other chronic pulmonary disease	241; 501; 523-526	241; 501; 523-526	490; 493; 515-518	490; 493-495; 500-508; 516; 517	J41; J42; J45-J47; J60-J70
	Rheumatic disease	710,0; 722,0; 722,1; 456	710,0; 722,0; 722,1; 456,0-456,3	446; 696,00; 712,0-712,3; 712,5; 716; 734	446; 696A; 710A-E; 714; 719D; 720; 725	M05; M06; M07.0-M07.3; M08; M12.3; M13; M30; M31.3-M31.6; M32; M33; M34; M35.0; M35.1; M35.3; M45-M46
	Alzheimer's disease				331A-C; 331X	G30; G31.1; G31.9
	Hemiplegia, tetraplegia	351; 352; 357	351; 352; 357,00	343; 344	342; 343; 344A-F	G11.4; G80; G81; G82; G83.0-G83.3; G83.8
	Diabetes	260	260	250	250	E10-E14
	Moderate or severe kidney disease	592; 593; 792	592; 593; 792	403; 404; 582; 583; 584; 593,0; 792; Y29,01	403; 582; 583; 585; 586; 588A; V42A; V45B; V56	I12.0; I13.1; N03.2-N03.7; N05.2-N05.7; N11; N18; N19; N25.0; Q61.1-Q61.4; Z49; Z94.0; Z99.2
	Liver disease	462,1; 581	462,1; 581	070; 456,0; 571; 573; 785,3	070; 456A; 456B; 456C; 571C; 571E; 571F; 572C-E; 573; 789F	B15-B19; I85; I98.2; I98.3; K70.3; K73; K74; K75.4; R18
	(Peptic) Ulcer disease	540-542	540-542	531-534	531-534	K25-K28
	Any malignancy	140-204; except 191	140-204; except 191	140-209; except 173; 208	140-208; except 173	C00-C97; except C87
	HIV/AIDS				079J; 279K	B20-B24; O98.7; R75; Z11.4; Z21; Z71.7
Suicide attempt		E970-E979	E971-E979	E950-E959	E950-E959	X60-X84; Y10-Y34

Table S3 Distribution of healthcare workers based on occupational qualification level (N=757,909)

Occupational qualification level	Occupations	n (%)
High-qualified (N=243,183)		
	Physicians	32,404 (13.3)
	Registered nurses	106,600 (43.8)
	Dentists and dental hygienists	9,077 (3.7)
	Psychologists, psychotherapists, counsellors	11,848 (4.9)
	Pharmacists and prescriptionists	6,297 (2.6)
	Therapists and other allied health professionals	33,730 (13.9)
	Healthcare administrators (high-qualified)	43,227 (17.8)
Low-qualified (N=514,726)		
	Assistant nurses	495,130 (96.2)
	Healthcare administrators (low-qualified)	19,596 (3.8)

Table S4 Demographic, health, and socioeconomic characteristics of the study population (N=4,573,875), based on suicide and suicide attempt outcomes for each occupational qualification level

**Variables**	**Categories**				**Suicide**				**Suicide attempt**
			**High-qualified**		**Low-qualified**		**High-qualified**		**Low-qualified**
		**Yes**	**No**	**Yes**	**No**	**Yes**	**No**	**Yes**	**No**
		**N=2,764**	**N=2,029,495**	**N=6,614**	**N=2,535,002**	**N=18,692**	**N=2,013,567**	**N=48,477**	**N=2,493,139**
		**n (%)**	**n (%)**	**n (%)**	**n (%)**	**n (%)**	**n (%)**	**n (%)**	**n (%)**
Follow-up time (years)	Mean (SD)	7.1 (4.3)	11.9 (4.6)	7.1 (4.2)	12.5 (4.2)	5.5 (3.7)	11.9 (4.6)	5.3 (3.8)	12.5 (4.2)
	Median (IQR)	7.0 (3.4-10.8)	15.0 (8.8-15.0)	7.0 (3.5-10.7)	15.0 (11.5-15.0)	5.0 (2.4-8.4)	15.0 (8.7-15.0)	4.6 (2.1-8.1)	15.0 (11.4-15.0)
Sex	Female	977 (35.4)	1,074,983 (53.0)	1,829 (27.7)	1,214,677 (47.9)	10,124 (54.2)	1,065,836 (52.9)	22,364 (46.1)	1,194,142 (47.9)
	Male	1,787 (64.7)	954,512 (47.0)	4,785 (72.4)	1,320,325 (52.1)	8,568 (45.8)	947,731 (47.1)	26,113 (53.9)	1,298,997 (52.1)
Birth year	1941-1950	338 (12.2)	512,918 (25.3)	526 (8.0)	463,385 (18.3)	2,289 (12.3)	510,967 (25.4)	2,995 (6.2)	460,916 (18.5)
	1951-1960	937 (33.9)	502,648 (24.8)	1,702 (25.7)	496,191 (19.6)	5,308 (28.4)	498,277 (24.8)	8,481 (17.5)	489,412 (19.6)
	1961-1970	950 (34.4)	547,693 (27.0)	1,931 (29.2)	566,861 (22.4)	6,042 (32.3)	542,601 (27.0)	12,421 (25.6)	556,371 (22.3)
	1971-1980	471 (17.0)	418,771 (20.6)	1,546 (23.4)	565,360 (22.3)	4,250 (22.7)	414,992 (20.6)	13,379 (27.6)	553,527 (22.2)
	1981-1989	68 (2.5)	47,465 (2.3)	909 (13.7)	443,205 (17.5)	803 (4.3)	46,730 (2.3)	11,201 (23.1)	432,913 (17.4)
Age	Mean (SD)	43.7 (9.4)	45.2 (11.4)	39.2 (11.1)	40.1 (13.4)	42.2 (10.2)	45.2 (11.4)	35.8 (11.4)	40.2 (13.4)
Foreign background	No	2,417 (87.5)	1,819,775 (89.7)	5,585 (84.4)	2,108,665 (83.2)	16,411 (87.8)	1,805,781 (89.7)	40,197 (82.9)	2,074,053 (83.2)
	Yes	347 (12.6)	209,720 (10.3)	1,029 (15.6)	426,337 (16.8)	2,281 (12.2)	207,786 (10.3)	8,280 (17.1)	419,086 (16.8)
History of mental disorders	No	1,847 (66.8)	1,923,972 (94.8)	4,072 (61.6)	2,322,590 (91.6)	14,402 (77.1)	1,911,417 (94.9)	33,377 (68.9)	2,293,285 (92.0)
	Yes	917 (33.2)	105,523 (5.2)	2,542 (38.4)	212,412 (8.4)	4,290 (23.0)	102,150 (5.1)	15,100 (31.2)	199,854 (8.0)
History of severe somatic disorders	No	2,375 (85.9)	1,818,854 (89.6)	5,595 (84.6)	2,274,005 (89.7)	16,110 (86.2)	1,805,119 (89.7)	41,837 (86.3)	2,237,763 (89.8)
	Yes	389 (14.1)	210,641 (10.4)	1,019 (15.4)	260,997 (10.3)	2,582 (13.8)	208,448 (10.4)	6,640 (13.7)	255,376 (10.2)
History of suicide attempts	No	2,419 (87.5)	2,004,893 (98.8)	5,536 (83.7)	2,476,209 (97.7)	16,520 (88.4)	1,990,792 (98.9)	40,390 (83.3)	2,441,355 (97.9)
	Yes	345 (12.5)	24,602 (1.2)	1,078 (16.3)	58,793 (2.3)	2,172 (11.6)	22,775 (1.1)	8,087 (16.7)	51,784 (2.1)
Parental socioeconomic positions in childhood	Unskilled manual	539 (19.5)	373,189 (18.4)	1,850 (28.0)	679,213 (26.8)	3,585 (19.2)	370,143 (18.4)	13,514 (27.9)	667,549 (26.8)
	Skilled manual	564 (20.4)	414,549 (20.4)	1,786 (27.0)	637,458 (25.2)	3,940 (21.1)	411,173 (20.4)	12,644 (26.1)	626,600 (25.1)
	Assistant non-manual	331 (12.0)	261,254 (12.9)	590 (8.9)	217,275 (8.6)	2,370 (12.7)	259,215 (12.9)	4,224 (8.7)	213,641 (8.6)
	Intermediate non-manual	636 (23.0)	473,934 (23.4)	875 (13.2)	330,215 (13.0)	4,205 (22.5)	470,365 (23.4)	6,184 (12.8)	324,906 (13.0)
	Higher non-manual	278 (10.1)	198,999 (9.8)	250 (3.8)	96,769 (3.8)	1,697 (9.1)	197,580 (9.8)	1,779 (3.7)	95,240 (3.8)
	Farmer	106 (3.8)	97,554 (4.8)	287 (4.3)	140,444 (5.5)	788 (4.2)	96,872 (4.8)	1,772 (3.7)	138,959 (5.6)
	No registered occupation	310 (11.2)	210,016 (10.4)	976 (14.8)	433,628 (17.1)	2,107 (11.3)	208,219 (10.3)	8,360 (17.3)	426,244 (17.1)

Table S5 Distribution of suicide methods by occupations (N=9,378)

Occupational qualification level	Occupations	Poisoning due to drugs n (%)	Poisoning due to other substances n (%)	Drowning and suffocation[a] n (%)	Firearms and explosives n (%)	Burns n (%)	Cutting or piercing n (%)	Others n (%)
High-qualified								
	Physicians	25 (36.2)	-[b]	20 (29.0)	-[b]	-[b]	-[b]	12 (17.4)
	Registered nurses	100 (56.5)	5 (2.8)	46 (26.0)	-[b]	-[b]	-[b]	17 (9.6)
	Other healthcare workers	29 (31.9)	-[b]	40 (44.0)	-[b]	-[b]	5 (5.5)	13 (14.3)
	Healthcare administrators (high-qualified)	19 (39.6)	-[b]	19 (39.6)	-[b]	-[b]	-[b]	6 (12.5)
	Non-healthcare workers (high-qualified)	617 (25.9)	84 (3.5)	1,004 (42.2)	173 (7.3)	58 (2.4)	78 (3.3)	365 (15.3)
Low-qualified								
	Assistant nurses	543 (46.8)	31 (2.7)	379 (32.7)	19 (1.6)	25 (2.2)	22 (1.9)	141 (12.2)
	Healthcare administrators (low-qualified)	10 (37.0)	-[b]	13 (48.2)	-[b]	-[b]	-[b]	-[b]
	Non-healthcare workers (low-qualified)	1,478 (27.2)	239 (4.4)	2,264 (41.7)	508 (9.4)	125 (2.3)	112 (2.1)	701 (12.9)

[a]Including hanging

[b]Estimates were not presented due to the low number of observations

Table S6 Distribution of first suicide attempt methods by occupations (N=67,169)

Occupational qualification level	Occupations	Poisoning due to drugs n (%)	Poisoning due to other substances n (%)	Drowning and suffocation[a] n (%)	Firearms and explosives n (%)	Burns n (%)	Cutting or piercing n (%)	Others n (%)
High-qualified								
	Physicians	88 (34.0)	6 (2.3)	5 (1.9)	-[b]	-[b]	22 (8.5)	136 (52.5)
	Registered nurses	542 (43.4)	37 (3.0)	7 (0.6)	-[b]	10 (0.8)	114 (9.1)	538 (43.1)
	Other healthcare workers	199 (34.5)	17 (3.0)	9 (1.6)	-[b]	-[b]	59 (10.2)	290 (50.3)
	Healthcare administrators (high-qualified)	191 (40.3)	18 (3.8)	-[b]	-[b]	-[b]	22 (4.6)	236 (49.8)
	Non-healthcare workers (high-qualified)	5,627 (34.9)	604 (3.7)	234 (1.5)	59 (0.4)	145 (0.9)	1,562 (9.7)	7,903 (49.0)
Low-qualified								
	Assistant nurses	6,264 (58.6)	476 (4.5)	142 (1.3)	18 (0.2)	64 (0.6)	875 (8.2)	2,852 (26.7)
	Healthcare administrators (low-qualified)	136 (51.1)	12 (4.5)	-[b]	-[b]	-[b]	25 (9.4)	89 (33.5)
	Non-healthcare workers (low-qualified)	15,460 (41.2)	1,912 (5.1)	715 (1.9)	167 (0.5)	312 (0.8)	3,957 (10.6)	14,997 (40.0)

[a]Including hanging
[b]Estimates were not presented due to the low number of observations

Table S7 Incidence rates of suicide and suicide attempt among physicians based on their specialties

Specialties			Suicide			Suicide attempt
	n	Follow-up (person-years)	incidence rate per 100,000 person-years (95% CI)	n	Follow-up (person-years)	incidence rate per 100,000 person-years (95% CI)
Non-licensed physicians	9	99,178.7	9.1 (4.7-17.4)	63	98,636.6	63.9 (49.9-81.8)
Licensed non-specialist physicians	-[a]	15,130.8	-[a]	11	15,055.2	73.1 (40.5-131.9)
Surgery, anesthesia, intensive care	15	48,699.9	30.8 (18.6-51.1)	44	48,353.0	91.0 (67.7-122.3)
Internal medicine	5	26,759.1	18.7 (7.8-44.9)	10	26,691.2	37.5 (20.2-69.6)
Pediatrics	-[a]	8,470.9	-[a]	-[a]	8,460.8	-[a]
General practice	15	48,749.8	30.8 (18.5-51.0)	44	48,484.9	90.7 (67.5-121.9)
Psychiatry	6	14,464.6	41.5 (18.6-92.3)	14	14,370.9	97.4 (57.7-164.5)
Radiology, clinical laboratory, other specialties	5	28,538.4	17.5 (7.3-42.1)	17	28,449.5	59.8 (37.1-96.1)
Other medical education, including PhD	9	61,918.0	14.5 (7.6-27.9)	43	61,621.6	69.8 (51.8-94.1)
Unknown	-[a]	22,328.4	-[a]	11	22,254.7	49.4 (27.4-89.3)

[a]Estimates were not presented due to the low number of observations

Table S8 Hazard ratios (95% CI) of suicide and suicide attempt among individuals born from 1972 onwards

Occupational qualification level	Occupations	Suicide HR (95% CI)			Suicide attempt HR (95% CI)		
		Model 1[a]	Model 2[b]	Model 3[c]	Model 1[a]	Model 2[b]	Model 3[c]
High-qualified							
	Physicians	-[d]	-[d]	-[d]	0.69 (0.50-0.95)	0.74 (0.54-1.03)	0.88 (0.64-1.23)
	Registered nurses	2.61 (1.83-3.71)	2.37 (1.67-3.38)	2.46 (1.73-3.50)	1.25 (1.09-1.43)	1.18 (1.03-1.35)	1.19 (1.04-1.36)
	Dentists and dental hygienists	-[d]	-[d]	-[d]	0.91 (0.55-1.52)	0.90 (0.54-1.50)	0.98 (0.59-1.62)
	Psychologists, psychotherapists, counsellors	-[d]	-[d]	-[d]	1.28 (0.78-2.09)	1.21 (0.74-1.98)	1.36 (0.83-2.23)
	Pharmacists and prescriptionists	-[d]	-[d]	-[d]	0.88 (0.47-1.63)	0.86 (0.46-1.60)	1.01 (0.54-1.88)
	Therapists and other allied health professionals	1.32 (0.65-2.66)	1.37 (0.68-2.78)	1.42 (0.70-2.88)	0.72 (0.55-0.93)	0.75 (0.58-0.97)	0.81 (0.62-1.05)
	Healthcare administrators (high-qualified)	-[d]	-[d]	-[d]	1.37 (1.11-1.69)	1.27 (1.03-1.57)	1.22 (0.99-1.50)
	Non-healthcare workers (high-qualified)	1	1	1	1	1	1
Low-qualified							
	Assistant nurses	1.54 (1.38-1.73)	1.32 (1.17-1.48)	1.30 (1.16-1.46)	1.32 (1.28-1.36)	1.17 (1.13-1.21)	1.11 (1.08-1.15)
	Healthcare administrators (low-qualified)	0.90 (0.48-1.68)	0.90 (0.49-1.68)	0.93 (0.50-1.73)	0.82 (0.68-0.98)	0.83 (0.69-1.00)	0.87 (0.72-1.05)
	Non-healthcare workers (low-qualified)	1	1	1	1	1	1

[a]Adjusted for sex, birth year, foreign background

[b]Adjusted for sex, birth year, foreign background, parental socioeconomic positions, history of mental disorders, history of somatic disorders, history of suicide attempts

[c]Adjusted for sex, birth year, foreign background, parental socioeconomic positions, history of mental disorders, history of somatic disorders, history of suicide attempts, school grades

[d]Estimates were not presented due to low number of observations

Table S9 Hazard ratios (95% CI) for suicide and suicide attempt among individuals working within public sectors

Occupational qualification level	Occupations	Suicide HR (95% CI)		Suicide attempt HR (95% CI)	
		Model 1[a]	Model 2[b]	Model 1[a]	Model 2[b]
High-qualified					
	Physicians	1.46 (1.10-1.94)	1.56 (1.17-2.08)	0.85 (0.74-0.98)	0.90 (0.78-1.04)
	Registered nurses	2.01 (1.67-2.42)	1.88 (1.56-2.26)	1.31 (1.22-1.41)	1.25 (1.17-1.34)
	Dentists and dental hygienists	-[c]	-[c]	1.16 (0.89-1.50)	1.19 (0.91-1.54)
	Psychologists, psychotherapists, counsellors	2.08 (1.28-3.36)	1.84 (1.14-2.99)	1.14 (0.92-1.42)	1.08 (0.87-1.34)
	Pharmacists and prescriptionists	1.66 (0.78-3.50)	1.63 (0.77-3.44)	1.03 (0.77-1.38)	1.03 (0.77-1.38)
	Therapists and other allied health professionals	1.36 (0.94-1.96)	1.33 (0.92-1.93)	1.06 (0.93-1.21)	1.07 (0.94-1.22)
	Healthcare administrators (high-qualified)	1.34 (0.97-1.86)	1.23 (0.89-1.71)	1.34 (1.21-1.49)	1.26 (1.13-1.40)
	Non-healthcare workers (high-qualified)	1	1	1	1
Low-qualified					
	Assistant nurses	1.72 (1.52-1.94)	1.56 (1.38-1.76)	1.27 (1.22-1.32)	1.18 (1.13-1.23)
	Healthcare administrators (low-qualified)	0.77 (0.46-1.28)	0.74 (0.44-1.24)	0.90 (0.77-1.06)	0.89 (0.76-1.04)
	Non-healthcare workers (low-qualified)	1	1	1	1

[a]Adjusted for sex, birth year, foreign background
[b]Adjusted for sex, birth year, foreign background, parental socioeconomic positions, history of mental disorders, history of somatic disorders, history of suicide attempts
[c]Estimates were not presented due to the low number of observations

Table S10 Number of cases, follow-up time, incidence rates (95% CI), and hazard ratios (95% CI) for suicide attempt by occupations, among individuals with no previous history of suicide attempts

Occupational qualification level	Occupations	n	Follow-up (person-years)	Incidence rate per 100,000 person-years (95% CI)	HR (95% CI) Model 1[a]	HR (95% CI) Model 2[b]
High-qualified						
	Physicians	242	368,945.8	65.6 (57.8-74.4)	0.94 (0.82-1.06)	0.97 (0.86-1.11)
	Registered nurses	1,071	1,255,671.6	85.3 (80.3-90.6)	1.29 (1.21-1.37)	1.26 (1.19-1.35)
	Dentists and dental hygienists	71	102,789.7	69.1 (54.7-87.2)	1.01 (0.80-1.27)	1.05 (0.83-1.33)
	Psychologists, psychotherapists, counsellors	93	126,483.4	73.5 (60.0-90.1)	1.09 (0.89-1.34)	1.08 (0.88-1.33)
	Pharmacists and prescriptionists	47	65,328.8	71.9 (54.1-95.8)	1.06 (0.80-1.42)	1.07 (0.80-1.42)
	Therapists and other allied health professionals	296	413,542.9	71.6 (63.9-80.2)	1.07 (0.96-1.21)	1.08 (0.96-1.21)
	Healthcare administrators (high-qualified)	415	489,961.0	84.7 (76.9-93.3)	1.25 (1.13-1.38)	1.21 (1.10-1.33)
	Non-healthcare workers (high-qualified)	14,285	20,879,625.6	68.4 (67.3-69.5)	1	1
Low-qualified						
	Assistant nurses	8,402	5,925,585.9	141.8 (138.8-144.9)	1.23 (1.20-1.26)	1.17 (1.14-1.20)
	Healthcare administrators (low-qualified)	220	230,099.5	95.6 (83.8-109.1)	0.80 (0.70-0.92)	0.81 (0.71-0.92)
	Non-healthcare workers (low-qualified)	31,768	24,528,178.4	129.5 (128.1-130.9)	1	1

[a]Adjusted for sex, birth year, foreign background

[b]Adjusted for sex, birth year, foreign background, parental socioeconomic positions, history of mental disorders, history of somatic disorders

Table S11 Hazard ratios (95% CI) for suicide and suicide attempt with later start of follow-up (30th birthday or 1 January 2006)

Occupational qualification level	Occupations	Suicide HR (95% CI) Model 1[a]	Suicide HR (95% CI) Model 2[b]	Suicide attempt HR (95% CI) Model 1[a]	Suicide attempt HR (95% CI) Model 2[b]
High-qualified					
	Physicians	1.54 (1.22-1.94)	1.58 (1.25-2.00)	0.89 (0.79-1.01)	0.93 (0.82-1.05)
	Registered nurses	1.78 (1.53-2.08)	1.65 (1.41-1.92)	1.31 (1.24-1.39)	1.24 (1.17-1.31)
	Dentists and dental hygienists	0.95 (0.51-1.76)	0.99 (0.53-1.85)	1.00 (0.80-1.24)	1.03 (0.83-1.28)
	Psychologists, psychotherapists, counsellors	1.72 (1.13-2.63)	1.60 (1.05-2.44)	1.07 (0.89-1.29)	1.03 (0.86-1.24)
	Pharmacists and prescriptionists	1.34 (0.64-2.81)	1.40 (0.67-2.95)	1.09 (0.84-1.41)	1.14 (0.88-1.48)
	Therapists and other allied health professionals	1.55 (1.18-2.03)	1.55 (1.19-2.03)	1.06 (0.95-1.18)	1.07 (0.96-1.20)
	Healthcare administrators (high-qualified)	1.10 (0.83-1.48)	1.03 (0.77-1.38)	1.25 (1.14-1.38)	1.19 (1.08-1.30)
	Non-healthcare workers (high-qualified)	1	1	1	1
Low-qualified					
	Assistant nurses	1.44 (1.33-1.55)	1.29 (1.19-1.39)	1.27 (1.23-1.30)	1.16 (1.13-1.19)
	Healthcare administrators (low-qualified)	0.61 (0.39-0.95)	0.61 (0.39-0.94)	0.81 (0.71-0.93)	0.82 (0.71-0.94)
	Non-healthcare workers (low-qualified)	1	1	1	1

[a]Adjusted for sex, birth year, foreign background

[b]Adjusted for sex, birth year, foreign background, parental socioeconomic positions, history of mental disorders, history of somatic disorders, history of suicide attempts

Table S12 Hazard ratios (95% CI) for suicide and suicide attempt with earlier end of follow-up (31 December 2010)

Occupational qualification level	Occupations	Suicide HR (95% CI)		Suicide attempt HR (95% CI)	
		Model 1[a]	Model 2[b]	Model 1[a]	Model 2[b]
High qualified					
	Physicians	1.89 (1.32-2.69)	1.99 (1.39-2.84)	1.05 (0.89-1.23)	1.13 (0.96-1.33)
	Registered nurses	1.90 (1.46-2.47)	1.68 (1.29-2.19)	1.39 (1.28-1.51)	1.29 (1.19-1.40)
	Dentists and dental hygienists	1.84 (0.87-3.87)	2.01 (0.95-4.24)	1.02 (0.75-1.39)	1.10 (0.81-1.49)
	Psychologists, psychotherapists, counsellors	2.39 (1.31-4.34)	2.09 (1.15-3.79)	1.05 (0.80-1.37)	1.00 (0.76-1.31)
	Pharmacists and prescriptionists	-[c]	-[c]	1.19 (0.84-1.69)	1.24 (0.88-1.76)
	Therapists and other allied health professionals	1.13 (0.65-1.96)	1.12 (0.65-1.94)	1.22 (1.05-1.41)	1.25 (1.08-1.45)
	Healthcare administrators (high qualified)	1.46 (0.96-2.22)	1.34 (0.88-2.04)	1.21 (1.06-1.38)	1.13 (0.99-1.29)
	Non-healthcare workers (high qualified)	1	1	1	1
Low qualified					
	Assistant nurses	1.30 (1.14-1.47)	1.10 (0.97-1.24)	1.28 (1.24-1.32)	1.14 (1.10-1.17)
	Healthcare administrators (low qualified)	-[c]	-[c]	0.80 (0.67-0.94)	0.81 (0.68-0.96)
	Non-healthcare workers (low qualified)	1	1	1	1

[a]Adjusted for sex, birth year, foreign background

[b]Adjusted for sex, birth year, foreign background, parental socioeconomic positions, history of mental disorders, history of somatic disorders, history of suicide attempts

[c]Estimates were not presented due to low number of observations

Table S13 Number of workers, number of cases, incidence rates (95% CI), and hazard ratios (95% CI) for suicide across occupations stratified by sex

Occupational qualification level	Occupations	Women N (n)	Incidence rate per 100,000 person-years (95% CI)	HR (95% CI) Model 1[a]	Model 2[b]	Men N (n)	Incidence rate per 100,000 person-years (95% CI)	HR (95% CI) Model 1[a]	Model 2[b]	p-value for interaction[c]
High-qualified										
	Physicians	14,409 (22)	12.5 (8.3–19.1)	1.75 (1.15–2.68)	1.79 (1.17–2.75)	17,995 (47)	23.6 (17.8–31.5)	1.40 (1.04–1.87)	1.49 (1.11–1.99)	0.24
	Registered nurses	97,373 (144)	12.3 (10.5–14.5)	1.75 (1.47–2.10)	1.67 (1.39–2.00)	9,227 (33)	27.9 (19.9–39.3)	1.80 (1.27–2.54)	1.44 (1.02–2.03)	
	Dentists and dental hygienists	6,339 (-[d])	-[d]	-[d]	-[d]	2,738 (9)	32.1 (16.7–61.7)	1.87 (0.97–3.61)	1.93 (1.00–3.72)	
	Psychologists, psychotherapists, counselors	9,217 (15)	15.0 (9.0–24.8)	2.06 (1.23–3.43)	1.93 (1.16–3.22)	2,631 (7)	24.2 (11.5–50.8)	1.45 (0.69–3.05)	1.21 (0.58–2.55)	
	Pharmacists and prescriptionists	5,859 (5)	8.1 (3.4–19.5)	1.16 (0.48–2.80)	1.21 (0.50–2.92)	438 (-[d])	-[d]	-[d]	-[d]	
	Therapists and other allied health professionals	28,580 (39)	10.9 (8.0–15.0)	1.61 (1.17–2.23)	1.66 (1.20–2.29)	5,150 (12)	18.7 (10.6–33.0)	1.18 (0.67–2.09)	1.13 (0.64–1.99)	
	Healthcare administrators (high-qualified)	35,931 (34)	8.1 (5.8–11.4)	1.13 (0.80–1.59)	1.05 (0.74–1.48)	7,296 (14)	16.7 (9.9–28.3)	1.05 (0.62–1.78)	0.95 (0.56–1.61)	
	Non-healthcare workers (high-qualified)	878,252 (716)	6.8 (6.3–7.3)	1	1	910,824 (1,663)	15.5 (14.8–16.3)	1		
Low-qualified										
	Assistant nurses	428,835 (827)	15.5 (14.5–16.6)	1.46 (1.33–1.60)	1.34 (1.22–1.47)	66,295 (333)	38.6 (34.7–43.0)	1.42 (1.27–1.59)	1.15 (1.02–1.28)	0.11
	Healthcare administrators (low-qualified)	14,028 (13)	7.7 (4.5–13.3)	0.73 (0.42–1.25)	0.76 (0.44–1.31)	5,568 (14)	20.3 (12.0–34.3)	0.72 (0.43–1.22)	0.68 (0.40–1.15)	
	Non-healthcare workers (low-qualified)	773,643 (989)	10.2 (9.6–10.9)	1	1	1,253,247 (4,438)	28.4 (27.5–29.2)	1	1	

[a]Adjusted for sex (except for sex-stratified estimates), birth year, foreign background

[b]Adjusted for sex (except for sex-stratified estimates), birth year, foreign background, parental socioeconomic positions, history of mental disorders, history of somatic disorders, history of suicide attempts

[c]Interaction between sex and occupations (p-value presented for Model 2)

[d]Estimates were not presented due to low number of observations

Table S14 Number of workers, number of cases, incidence rates (95% CI), and hazard ratios (95% CI) for suicide attempt across occupations stratified by sex

Occupational qualification level	Occupations	Women N (n)	Women Incidence rate per 100,000 person-years (95% CI)	Women HR (95% CI) Model 1[a]	Women HR (95% CI) Model 2[b]	Men N (n)	Men Incidence rate per 100,000 person-years (95% CI)	Men HR (95% CI) Model 1[a]	Men HR (95% CI) Model 2[b]	p-value for interaction[c]
High-qualified										
	Physicians	14,409 (120)	68.8 (57.5–82.3)	0.89 (0.74–1.06)	0.92 (0.77–1.10)	17,995 (139)	70.2 (59.5–82.9)	0.89 (0.76–1.06)	0.95 (0.81–1.13)	0.6
	Registered nurses	97,373 (1,110)	95.7 (90.3–101.5)	1.26 (1.18–1.34)	1.21 (1.13–1.29)	9,227 (138)	117.9 (99.7–139.3)	1.56 (1.32–1.85)	1.34 (1.13–1.59)	
	Dentists and dental hygienists	6,339 (59)	77.4 (60.0–99.9)	1.00 (0.77–1.29)	1.07 (0.82–1.38)	2,738 (21)	75.2 (49.0–115.3)	0.95 (0.62–1.46)	0.98 (0.64–1.50)	
	Psychologists, psychotherapists, counselors	9,217 (86)	86.3 (69.8–106.6)	1.12 (0.91–1.39)	1.08 (0.87–1.34)	2,631 (20)	69.5 (44.8–107.7)	0.90 (0.58–1.40)	0.83 (0.53–1.28)	
	Pharmacists and prescriptionists	5,859 (48)	78.4 (59.1–104.0)	1.01 (0.76–1.34)	1.04 (0.79–1.39)	438 (5)	104.4 (43.5–250.8)	1.32 (0.55–3.18)	1.20 (0.50–2.88)	
	Therapists and other allied health professionals	28,580 (281)	79.3 (70.6–89.1)	1.05 (0.93–1.18)	1.08 (0.96–1.22)	5,150 (57)	89.6 (69.1–116.2)	1.17 (0.90–1.52)	1.15 (0.89–1.50)	
	Healthcare administrators (high-qualified)	35,931 (406)	97.9 (88.8–107.9)	1.27 (1.15–1.40)	1.19 (1.08–1.32)	7,296 (68)	81.8 (64.5–103.8)	1.04 (0.82–1.32)	0.98 (0.77–1.24)	
	Non-healthcare workers (high-qualified)	878,252 (8,014)	76.5 (74.9–78.2)	1	1	910,824 (8,120)	76.3 (74.7–78.0)	1	1	
Low-qualified										
	Assistant nurses	428,835 (8,942)	170.2 (166.7–173.7)	1.27 (1.24–1.31)	1.16 (1.13–1.20)	66,295 (1,749)	206.1 (196.6–216.0)	1.27 (1.21–1.33)	1.10 (1.05–1.15)	0.11
	Healthcare administrators (low-qualified)	14,028 (177)	106.4 (91.8–123.3)	0.80 (0.69–0.93)	0.82 (0.71–0.96)	5,568 (89)	130.3 (105.9–160.4)	0.82 (0.67–1.01)	0.81 (0.66–1.00)	
	Non-healthcare workers (low-qualified)	773,643 (13,245)	138.4 (136.1–140.8)	1	1	1,253,247 (24,275)	157.1 (155.1–159.1)	1	1	

[a]Adjusted for sex (except for sex-stratified estimates), birth year, foreign background
[b]Adjusted for sex (except for sex-stratified estimates), birth year, foreign background, history of mental disorders, history of somatic disorders, history of suicide attempts, parental socioeconomic positions
[c]Interaction between sex and occupations (p-value presented for Model 2)

Supplementary figures

(A) Suicide

(1) High qualified

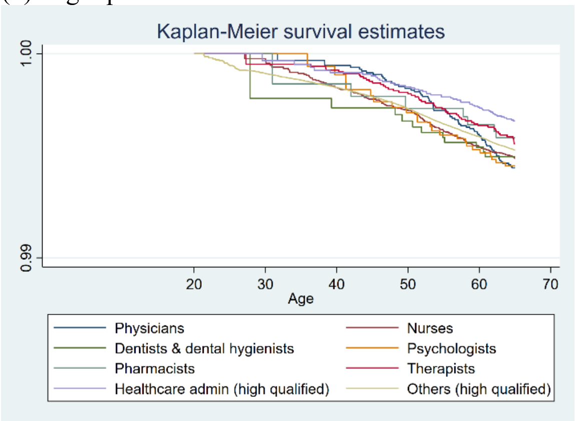

(2) Low qualified

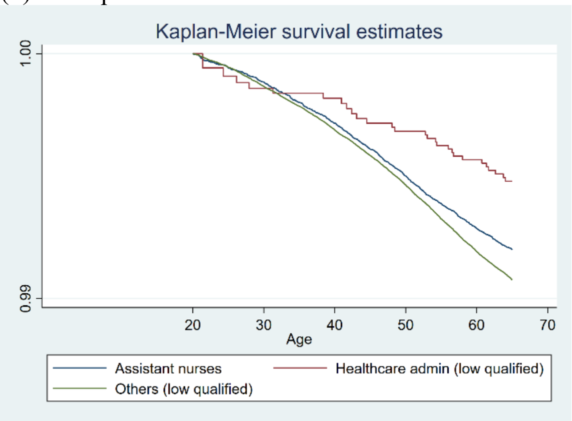

(B) Suicide attempt

(1) High qualified

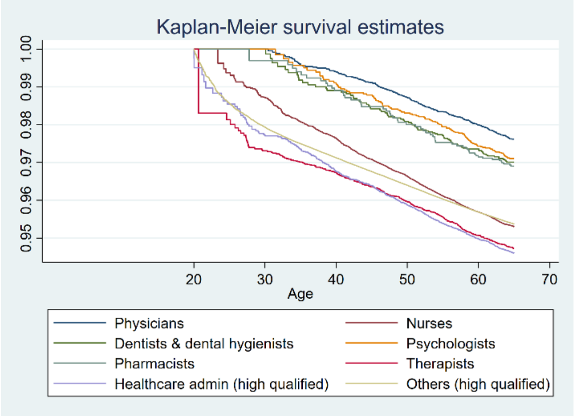

(2) Low qualified

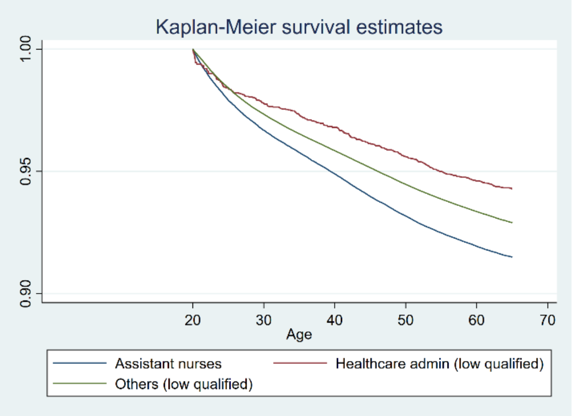

Figure S1 Kaplan-Meier curves for: (A) suicide and (B) suicide attempt among workers in (1) high qualified and (2) low qualified occupations.

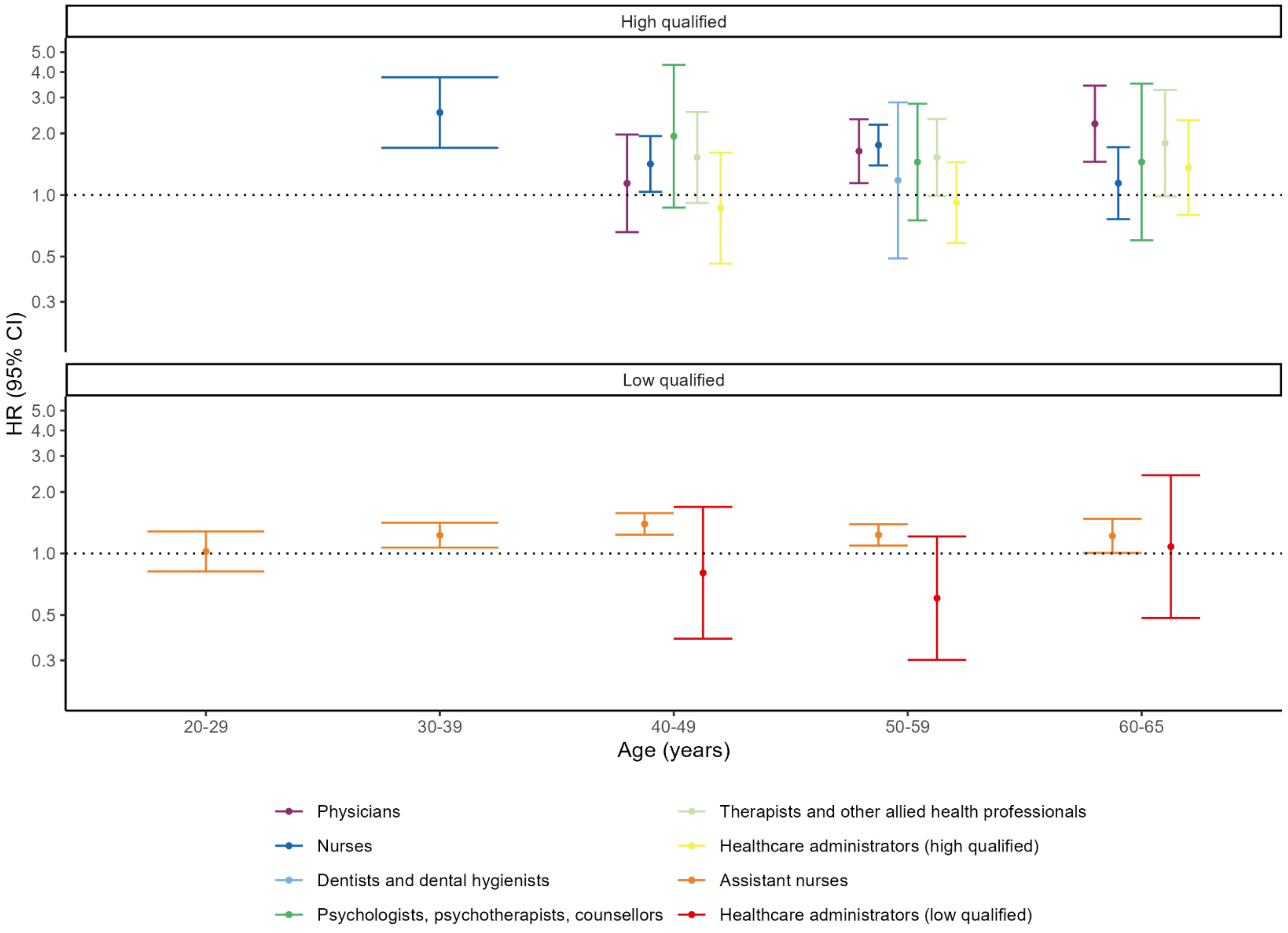

Figure S2 Hazard ratios (95% CI) for suicide by occupations stratified by age at follow up. Adjusted for birth year, foreign background, parental socioeconomic positions, history of mental disorders, history of somatic disorders, history of suicide attempts. Estimates with low number of observations were not presented. P-values for interaction between occupations and age are 0.55 (higher qualified) and 0.22 (lower qualified).

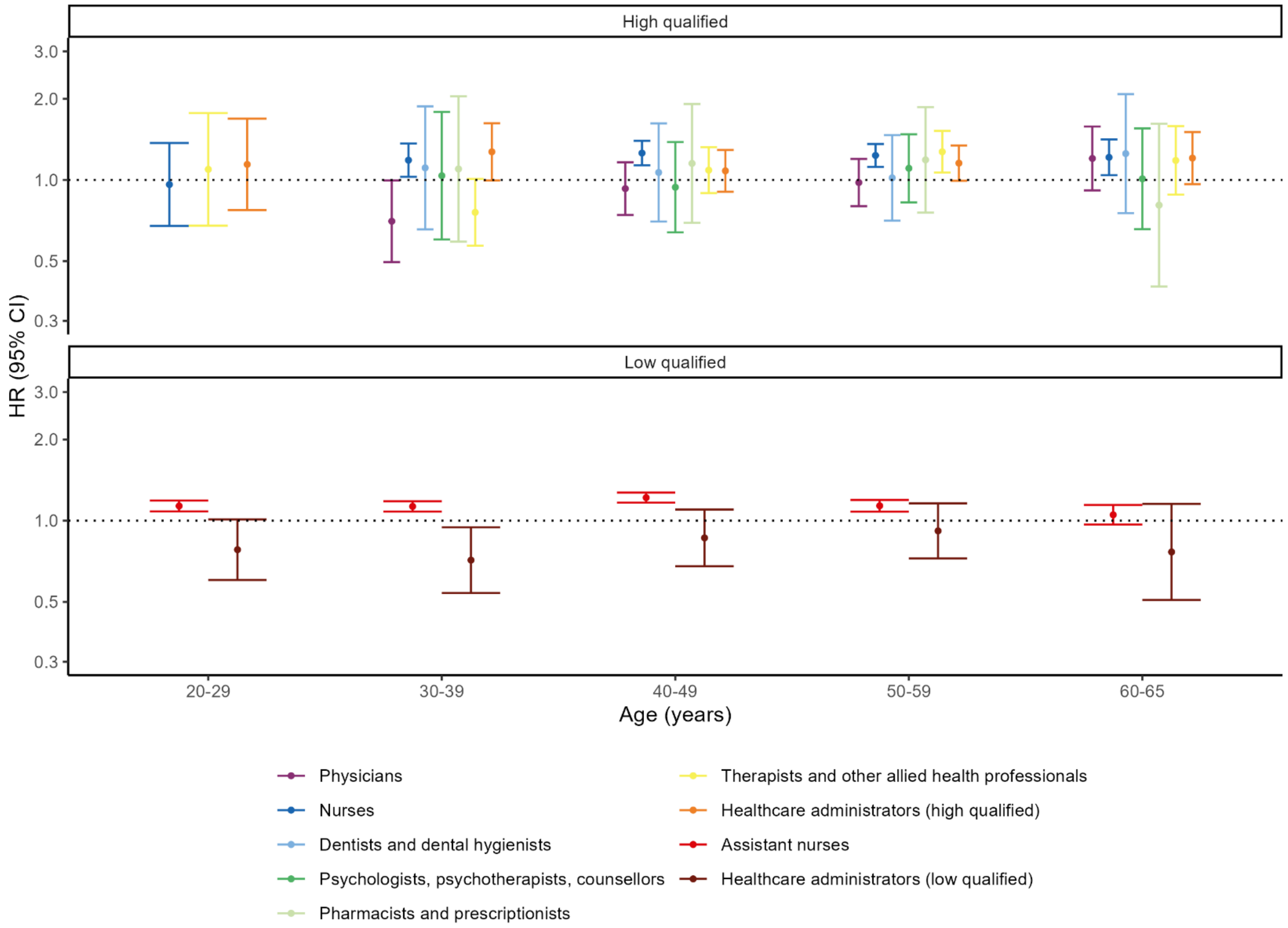

Figure S3 Hazard ratios (95% CI) for suicide attempt by occupations stratified by age at follow up. Adjusted for birth year, foreign background, parental socioeconomic positions, history of mental disorders, history of somatic disorders, history of suicide attempts. Estimates with low number of observations were not presented. P-values for interaction between occupations and age are 0.10 (higher qualified) and 0.08 (lower qualified).

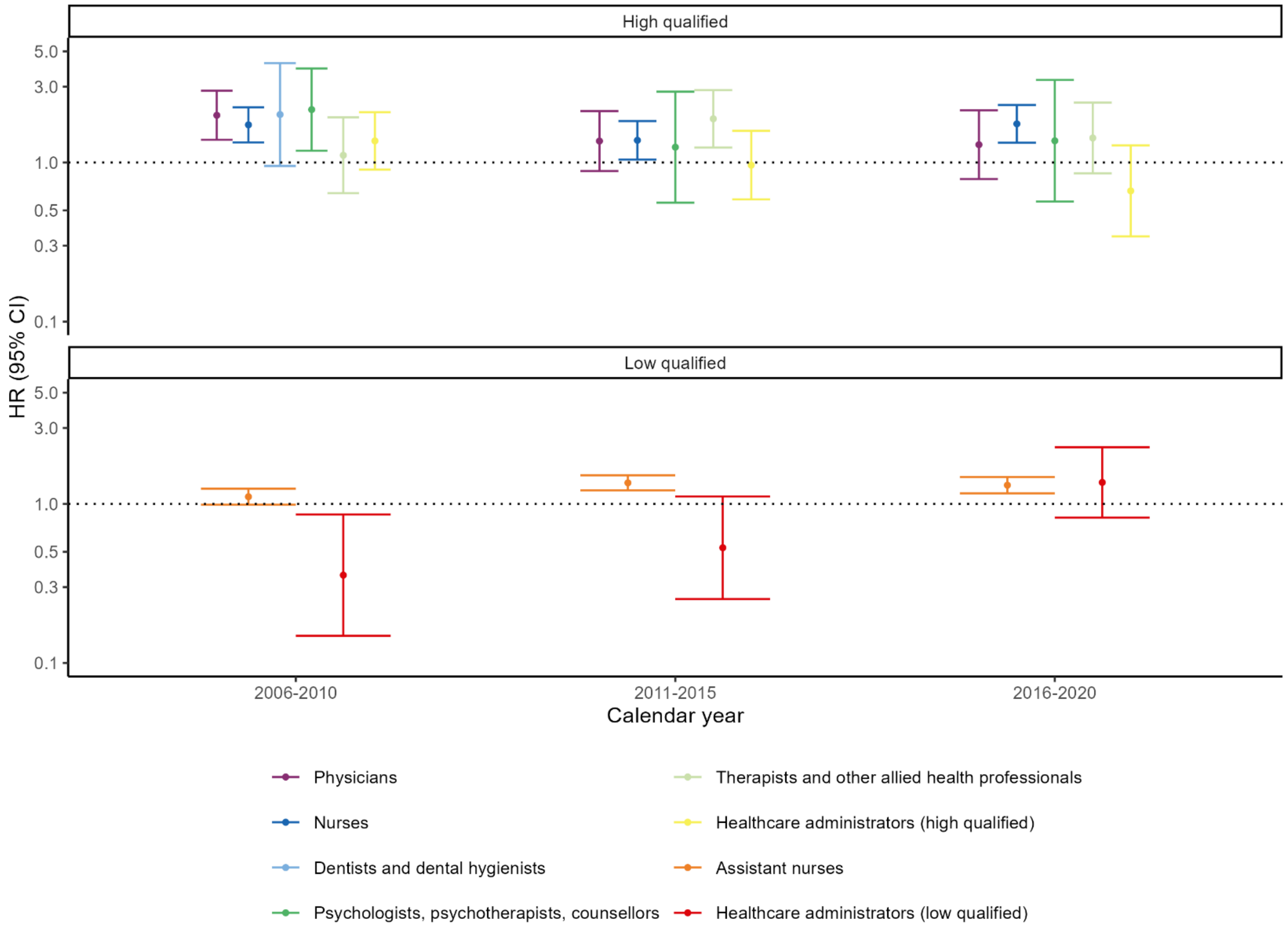

Figure S4 Hazard ratios (95% CI) for suicide by occupations stratified by calendar year. Adjusted for birth year, foreign background, parental socioeconomic positions, history of mental disorders, history of somatic disorders, history of suicide attempts. Estimates with low number of observations were not presented. P-values for interaction between occupations and age are 0.30 (higher qualified) and 0.00 (lower qualified).

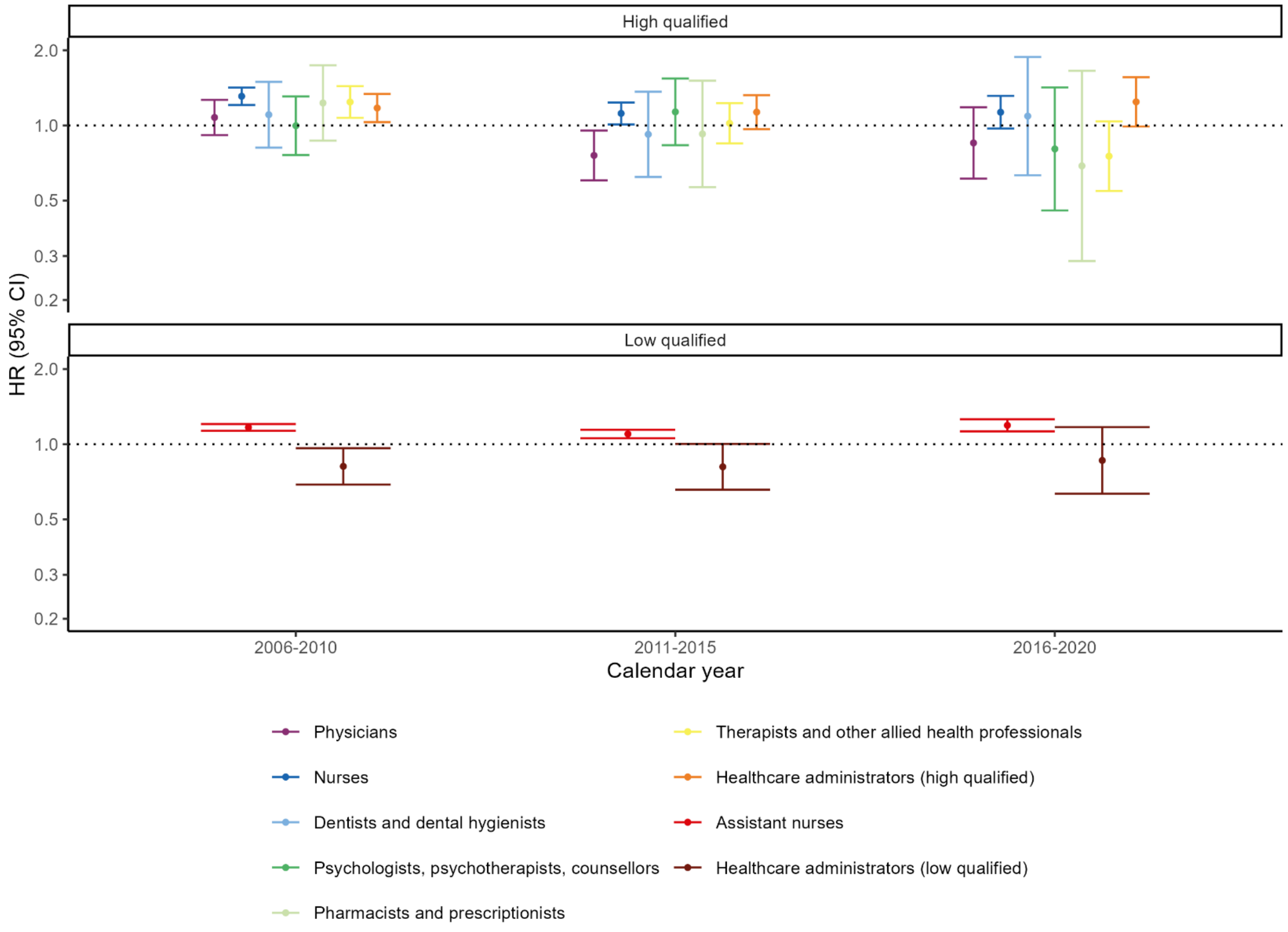

Figure S5 Hazard ratios (95% CI) for suicide attempt by occupations stratified by calendar year. Adjusted for birth year, foreign background, parental socioeconomic positions, history of mental disorders, history of somatic disorders, history of suicide attempts. Estimates with low number of observations were not presented. P-values for interaction between occupations and age are 0.02 (higher qualified) and 0.07 (lower qualified).

Supplementary references

1. Ludvigsson JF, Svedberg P, Olén O, Bruze G, Neovius M. The longitudinal integrated database for health insurance and labour market studies (LISA) and its use in medical research. Eur J Epidemiol. 2019 Apr 30;34(4):423–37.

2. Sörberg Wallin A, Sorjonen K, Lager A, Falkstedt D. Academic performance, subsequent socioeconomic status and suicide attempt in adulthood: path analyses on Swedish cohort data. J Epidemiol Community Health. 2020 Dec;74(12):1002–7.

3. Shen H, Magnusson C, Rai D, Lundberg M, Lê-Scherban F, Dalman C, et al. Associations of Parental Depression With Child School Performance at Age 16 Years in Sweden. JAMA Psychiatry. 2016;73(3):239.

4. Hagqvist E. Kan vi lita på den officiella statistiken om antalet läkare i Sverige? [Internet]. Läkartidningen. 2021 [cited 2023 Apr 6]. Available from: https://lakartidningen.se/opinion/debatt/2021/06/kan-vi-lita-pa-den-officiella-statistiken-om-antalet-lakare-i-sverige/

5. Hanson LLM, Nyberg A, Mittendorfer-Rutz E, Bondestam F, Madsen IEH. Work related sexual harassment and risk of suicide and suicide attempts: prospective cohort study. BMJ. 2020 Sep 2;370:m2984.
